# Indicators for palliative care integration in primary health care: how expert profile shapes consensus in a modified Delphi study

**DOI:** 10.1186/s12913-026-14781-y

**Published:** 2026-06-08

**Authors:** Carolina Muñoz Olivar, Juan Sebastián Gómez Quintero, Fernando Pastrana Rendon, Carla Taramasco Toro, Miguel Antonio Sánchez-Cárdenas, Carlos Javier Avendaño-Vásquez

**Affiliations:** 1 Health Sciences, Faculty of Medicine, Antonio Nariño University, Bogotá, Colombia; 2 Faculty of Engineering, Andrés Bello University, Santiago, Chile; 3 Faculty of Engineering, Institute of Technologies for Innovation in Health and Well-Being, Andrés Bello University, Viña del Mar, Chile; 4 Center for Cancer Prevention and Control (CECAN), Santiago, Chile; 5 Faculty of Nursing, El Bosque University, Bogotá, Colombia; 6 Escuela de enfermería, Universidad Industrial de Santander, Bucaramanga, Colombia; 7 Faculty of Nursing (Postgraduate Programs), Andres Bello University, Santiago, Chile

**Keywords:** Delphi technique, Palliative care, Primary health care, Indicators, Self-organizing maps, Neural network

## Abstract

**Background:**

Integrating palliative care into primary health care requires clear, feasible, and valid indicators. Consensus-based methods such as Delphi are commonly used for this purpose, but they may overlook differences in how heterogeneous expert panels prioritize indicators. This study aimed to strengthen the methodological approach used to select indicators for assessing the integration of palliative care into primary health care. This study aimed to strengthen the methodological approach used to select indicators for assessing the integration of palliative care into primary health care.

**Methods:**

A two-round modified Delphi was conducted with 23 experts from different regions of Colombia. Each indicator was rated for relevance, feasibility, and validity. A priori consensus was defined as *≥*70% of ratings *≥*7/10 and IQR *≤*2. Global content validity indices (S-CVI) and Lawshe’s content validity ratio (CVR) were calculated as supportive evidence. Expert profile was quantified using the Metric for Assessing Knowledge and Understanding (MAKU) and used to stratify analyses, To explore data patterns, separate 2*×*2 SOMs were trained for Strategic and Operational profiles using normalized medians and robust coefficients of variation. Neurons with higher median scores and lower dispersion were interpreted as favorable.

**Results:**

Global S-CVI increased from 0.86 in Round 1 to 0.88 in Round 2. By dimension, relevance rose from 0.82 to 0.85, feasibility remained stable at 0.72, and validity increased from 0.62 to 0.71. Stratification by expert profile revealed meaningful differences in consensus patterns. SOM analysis showed both shared and differing prioritization patterns across profiles. After removing overlaps, a final set of eighteen indicators was retained from the dominant neurons of the profile-specific SOMs.

**Conclusion:**

Combining a modified Delphi with MAKU-based stratification and self-organizing maps provided a transparent and profile-sensitive approach to strengthen consensus-based indicator selection in a heterogeneous expert panel. Differences in expert profile influenced how indicators were prioritized. This approach supports more rigorous and context-aware selection of indicators for monitoring palliative care integration in primary health care.

**Supplementary Information:**

The online version contains supplementary material available at 10.1186/s12913-026-14781-y.

## Background

Palliative care (PC) is recognized by the World Health Organization (WHO) as an ethical responsibility of health systems and an essential component of Universal Health Coverage [1, 2]. To operationalize this commitment, the public health strategy for PC was articulated in 2007 [3–5], and in 2014 the World Health Assembly urged Member States to integrate PC across health services, with emphasis on Primary Health Care (PHC) [1, 4]. Despite these milestones, access and quality remain uneven, particularly in Latin America [6, 7].

PHC is the main vehicle for building equitable, people-centred health systems, which are essential to achieving the Sustainable Development Goals (SDGs) [8–11]. In this approach, PC is not a stand-alone service but a core function to be delivered across the life course and levels of care [12, 13]. This integrated approach requires operationalizing PC at the primary care level. Primary Palliative Care (PPC) refers to PC provided by non-specialists at the first point of contact and in community settings, with timely collaboration with specialized PC when needed [14].

International frameworks consistently highlight the enabling roles of policy, workforce training, access to essential medicines, service delivery models, and research capacity [9, 15–17]. Yet national and subnational implementation is heterogeneous, and large gaps persist [6, 18].

Monitoring the integration of PC within a PHC approach requires indicators that capture both PC development (e.g., policy, medicines, training, service provision, research, empowered communities) and PHC system performance (e.g., integrated services, multisectoral action, and people- and community-orientation mapped across inputs, processes/outputs, and outcomes/impact) [12, 13]. However, comparable tools to assess integration across diverse territorial and organizational contexts are limited [6, 18].

Consensus-based methods are widely used to design and prioritize health indicators. The Delphi technique, which involves iterative and anonymous surveys with structured feedback, has an established record in health research, provided that expert selection, round-to-round transparency, and reporting standards are clear [19, 20]. However, these approaches often assume that expert panels are homogeneous and may overlook differences in how experts evaluate complex constructs. Variability in expert profiles can introduce noise and mask subgroup differences in judgment [21, 22]. To address this limitation, this study combines a modified Delphi with two complementary strategies: (i) the Metric for Assessing Knowledge and Understanding (MAKU) to evaluate panel quality and stratify experts within an already eligible panel, and (ii) self-organizing maps (SOMs) to identify and cluster indicators based on their median performance and robust dispersion, helping to detect stable patterns that complement a priori Delphi thresholds [23, 24]. Although consensus-based approaches such as the Delphi method are widely used to develop health indicators, they often assume that expert panels are homogeneous and may overlook differences in how experts evaluate complex constructs. This limitation can reduce the transparency of the consensus process and mask profile-dependent variations in judgment.

To address this gap, this study proposes a methodological approach that integrates expert consensus with profile-based stratification using the Metric for Assessing Knowledge and Understanding (MAKU), and pattern recognition using self-organizing maps (SOMs). The main aim was to strengthen the methodological approach used to select indicators for assessing the integration of palliative care into primary health care.

## Methods

### Study aim

The main aim of this study was to strengthen the methodological approach used to select indicators for assessing the integration of palliative care into primary health care. Specifically, this study combined expert consensus methods with profile-based stratification using the Metric for Assessing Knowledge and Understanding (MAKU), and pattern recognition using self-organizing maps (SOMs). As secondary objectives, the study aimed: (i) to identify a set of indicators through a modified Delphi process, and (ii) to examine how differences in expert profiles influence consensus patterns.

### Study design

We conducted a two-round modified Delphi process to reach consensus on indicators for measuring PC integration in PHC. The Delphi method achieves consensus among experts through repeated, anonymous surveys with structured feedback between rounds, allowing participants to refine their judgments until stability in responses is reached [25, 26].

**Fig. 1:**
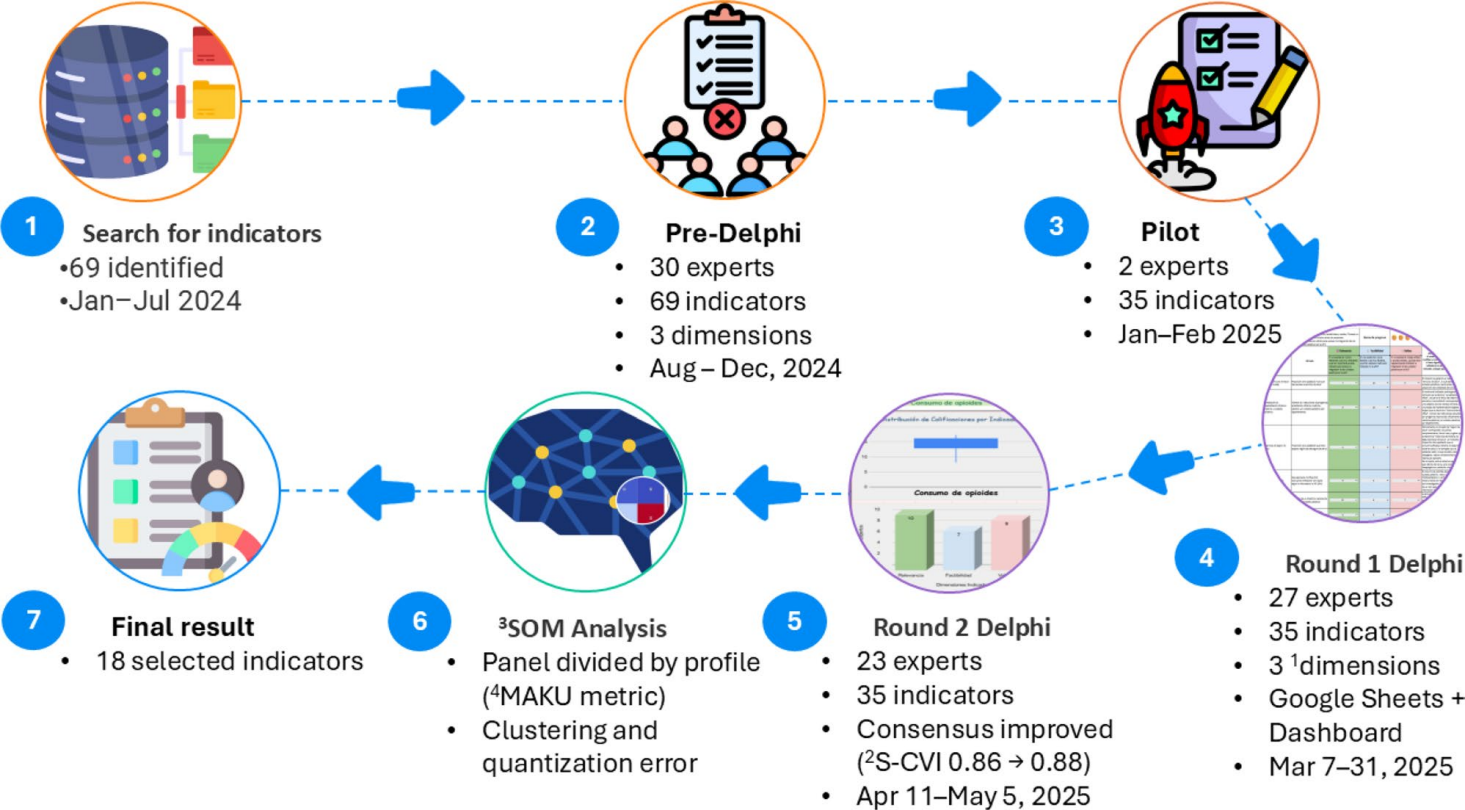
Modified Delphi process. *Legend:* Modified Delphi process to identify indicators for the integration of palliative care in primary health care. ^1^The evaluated dimensions were relevance, feasibility, and validity. ^2^CVI = content validity Index; ^3^SOM = self-organizing maps, an unsupervised neural network used for clustering and classification consistency; ^4^MAKU = metric for Assessing Knowledge and Understanding (used to evaluate panel quality and to stratify experts into two profiles: strategic and Operational. See steps 1–7

The modified Delphi is recognized as a flexible variation of the classical method, often used for the construction and validation of indicators because it reduces panel attrition and participant fatigue while maintaining rigour [27–29]. Considering the broad and heterogeneous set of indicators to be validated, we adopted a modified two-round Delphi to balance feasibility and efficiency with methodological rigour and efficiency [30–35]. The study adhered to the CREDES recommendations for reporting Delphi studies in palliative care [20]. The Fig. 1 illustrates the modified Delphi process.

### Indicator identification and pre-Delphi screening

An initial list of 69 candidate indicators was compiled from Colombian health system databases: National Administrative Department of Statistics (DANE) [36], High-Cost Account (CAC) [37], Ministry of Health Open Data platform (OTIC) [38], and the Colombian Palliative Care Observatory (OCCP) [39], and from peer-reviewed scientific literature and technical reports [12, 13]. (see Supplementary Table 1).

Two investigators (CMO and CAV) independently reviewed and refined the list, resolving discrepancies by discussion. To reduce cognitive burden in the main Delphi, a structured pre-Delphi was conducted in which 30 domain experts rated the 69 indicators. Based on these results, 35 indicators were retained for the two Delphi rounds. The detailed computational process used for pre-Delphi clustering and indicator selection, including self-organizing map training and expert stratification, is available in the companion repository [40].

### Expert panel

Experts were invited by email through professional associations, universities, health institutions, national and regional health authorities, and PC networks. Snowball sampling was also used. Eligible participants were health professionals with at least one year of experience in PC, PHC, or public health. In addition, participants had to meet at least one of the following: membership in a national PC association; employment at a higher-education institution; work in the Ministry of Health or regional health authority related to PC; involvement in public health policy; or service as national delegate to WHO/PAHO. The entire Delphi process was conducted in Spanish.

#### Profiles according to the MAKU metric

Given the intentional heterogeneity of the panel, and the absence of standardized metrics to assessing expert panel quality, we developed the Metric for Assessing Knowledge and Understanding (MAKU). This index has two aims: (i) to evaluate panel quality before initiating a Delphi study, and (ii) to stratify an already eligible panel. It is not intended to exclude participants. Its goal is to distinguish between Strategic and Operational Profiles without allowing differences in seniority to obscure these distinctions. MAKU is a formative, multidimensional metric that reflects relative profiles within the panel, rather than a diagnostic or exclusionary tool. Its purpose is to support sensitivity analyses of consensus by profile and to enhance transparency in Delphi-based decision making.

Four criteria frequently cited in the literature on expert selection were included: professional recognition, years of experience, highest academic degree, and publications [29, 34, 41–44]. Two independent investigators discussed these criteria and set the weighting scheme. Table 1 shows the scoring rubric.

**Table 1:** Evaluator profile categories and scoring

Professional Recognition	Years of Experience	Education Level	Publications
Not recognized (1)	Beginner (1–2 years) (1)	Undergraduate (1)	None or few (0–2) (1)
Moderately recognized (2)	Intermediate (2–5 years) (2)	Specialist (2)	Some relevant (3–5) (2)
Influential figure or reference (3)	Expert (* > 5*years) (3)	Master’s (3)	Many (*≥*6) (3)
		PhD (4)	

Each score was normalised to a 0–1 scale as follows:

$$\begin{aligned}I_n &= \frac{\mathrm{Influence}-1}{2}, &E_n &= \frac{\mathrm{Experience}-1}{2}, \\F_n &= \frac{\mathrm{Education}-1}{3}, &P_n &= \frac{\mathrm{Publications}-1}{2}.\end{aligned}$$

The individual MAKU metric for each participant was calculated as the mean of the four normalized dimensions. Scores below 0.40 were classified as Operational Profile and scores equal to or above 0.40 as Strategic Profile. For the overall panel quality assessment, we calculated the mean MAKU score across all experts in the panel. Quartiles were used to define thresholds: low (* < *0.375), moderate (0.375–0.625), and high (* > *0.625).

To validate the MAKU metric, descriptive analyses (range, mean, variance) were performed for the total panel and for each subgroup. Sensitivity analyses were conducted by varying the classification cut-off (0.35, 0.40, 0.45) to test the stability of assignments. Data distribution was examined with the Shapiro–Wilk test, and group differences were tested with Welch’s t-test. Robustness of the index was also evaluated by testing alternative weighting schemes (*±*10% points for each dimension). For each scheme, all experts (*n* = 23) were reclassified into Strategic or Operational Profile using the standard cut-off (0.40), and the resulting assignments were compared against the reference scheme of equal weights (25% each). The code and data are available at [40].

### Delphi questionnaire and procedures

A pilot test with two experts identified usability problems with online forms (fatigue and confusing presentation). Therefore, the Delphi questionnaire was implemented using Google Sheets with Apps Script automation. Each of the 35 indicators was rated on three dimensions: relevance, feasibility, and validity, using a 10-point scale (1 = very low, 10 = very high). Definitions of each dimension and the formula of each indicator were provided. Once submitted, responses were automatically locked, and an aggregated dashboard with descriptive statistics and graphs was displayed to the participant. The questionnaire used in the Delphi rounds (English version) is available as Supporting Information Supplementary File 1.

Round 1 was conducted from 7 to 31 March 2025, and Round 2 from 11 April–5 May 2025. Weekly reminders were sent to non-responders. In both rounds, experts rated all 35 indicators and were invited to provide optional free-text comments to support or explain their scores. Between rounds, participants received anonymised feedback. If consensus was reached, this was noted; if not, the most frequent rating and the percentage of experts who selected it were shown, together with each expert’s own Round 1 rating, allowing them to confirm or revise their score. Although free-text comments were not formally analyzed, we summarized recurring points descriptively to contextualize items that did not reach consensus.

### Consensus criteria

Consensus thresholds were defined a priori. An indicator was accepted if *≥*70% of experts rated it *≥*7 and the interquartile range (IQR) was *≤*2 (lower dispersion indicates higher agreement). Items not meeting both criteria were classified as “no consensus”. Consensus was analysed separately for each dimension.

For content validity, three indices were calculated [18, 45–50]:

- Content Validity Index (I-CVI):
  $$ CVI = \frac{\text{Number of experts rating item $\geq$ 7}}{\text{Total number of experts}} $$
  Indicators were considered acceptable with I-CVI *≥* 0.70.
- Scale-level Content Validity Index (S-CVI): Mean of item-level CVIs across a set of items (reported overall and by dimension).
- Content Validity Ratio (CVR): using Lawshe’s formula
  $$ CVR = \frac{n_e - N/2}{N/2} $$
  where $n_e$ = number of experts rating *≥*7, *N* = total panel size. The critical value of 0.39 for *N=23* was applied, and items with CVR *≥* 0.39 were considered valid.

### Data analysis and clustering

Only the experts who completed both Delphi rounds (*n* = 23) were included in the analysis. For each indicator, the three dimensions ratings (relevance, feasibility, validity) were combined into a single value for each expert using the Euclidean norm:

$$\mathrm{Norm}_i = \sqrt{r_i^2 + f_i^2 + v_i^2}$$

where $r_i$, $f_i$, and $v_i$ represent the ratings of relevance, feasibility, and validity, respectively.

This unidimensional summary was selected because the Euclidean norm preserves the relative contribution of each dimension while producing a scalar measure that reflects the overall magnitude of consensus. Unlike arithmetic averaging, this approach avoids compensatory effects between dimensions (e.g., a low value in one dimension being offset by a high value in another), and therefore provides a more balanced representation of the combined expert judgment across the three criteria. This property makes it particularly suitable for subsequent clustering and pattern recognition analyses [51]. Then, for each indicator, we calculated the median of these values across all experts, as well as a robust measure of variability: the robust coefficient of variation, defined as the interquartile range divided by the median (CV_r_ = IQR/median) [52, 53]. This measure was preferred over the conventional coefficient of variation because it relies on the median and IQR, which are less sensitive to extreme values and skewed distributions, providing a more stable estimate of variability in non-normal data. These two statistics (the median score and CV_r_) were used as input features for the clustering procedure.

We applied SOMs, a type of unsupervised neural network that groups similar inputs through competitive learning. Each indicator is assigned to a “neuron” based on its similarity to other indicators. Before training, the data were normalized based on their distribution to ensure comparability in the SOM Algorithm, which is sensitive to the scale of input values (variables with normal distribution (as assessed by the Shapiro–Wilk test) were scaled using StandardScaler, while non-normally distributed variables were scaled using RobustScaler). During training, the neurons adjust their values to better represent the inputs, and nearby neurons influence each other through a neighborhood function that shrinks over time. This process allows the map to organize indicators with similar profiles into nearby neurons [23, 24].

To explore potential differences in how indicators were rated by experts with different profiles, we trained two separate SOMs: one for the Strategic Profile subgroups and one for the Operational Profile subgroup, as defined by the MAKU metric. Each SOM used the two input features per indicator: the normalized median (median norm) and the robust coefficient of variation (CV_r_). We used a small grid 2*×*2 grid for parsimony and interpretability, with fixed parameters and a random seed to ensure reproducibility.

Training was performed using 10,000 iterations. The learning rate and neighborhood radius (*σ*) were reduced gradually over time. For the Strategic Profile SOM, the learning rate decreased from 0.002 to 0.0001 and *σ* from 0.01 to 0.003. For the Operational Profile SOM, the learning rate decreased from 0.001 to 0.00001 and *σ* from 0.5 to 0.2. All analyses were conducted in Python using JupyterLab, and all code is available in the public repository [54].

### Bias mitigation

We mitigated potential biases through pre-specified thresholds and stopping rules, sensitivity analyses, profile stratification with the MAKU metric (Strategic and Operational), and complementary SOM clustering. For indicators not aligned across profiles, phased implementation with predefined review points was preferred over immediate exclusion.

- **Selection bias:** broad invitations across institutions and snowball sampling.
- **Dominance bias:** anonymity of ratings and comments.
- **Fatigue bias:** pre-Delphi filtering, pilot testing, simplified interface, automated dashboards.
- **Attrition bias:** short reminders with concise, motivating messages.
- **Data loss bias:** automatic cloud storage and backup copies.

### Ethical considerations

This study was conducted in accordance with the ethical principles of the Declaration of Helsinki. Ethical approval was granted by the Bioethics Committee of the Faculty of Medicine at Antonio Nariño University (Colombia), under Act ${\textrm{N}^{\circ}}$ 13–2024, dated August 21, 2024. All participants provided informed consent before participating. Participation was voluntary, and all data were anonymized.

## Results

### Flow and panel characteristics

**Fig. 2:**
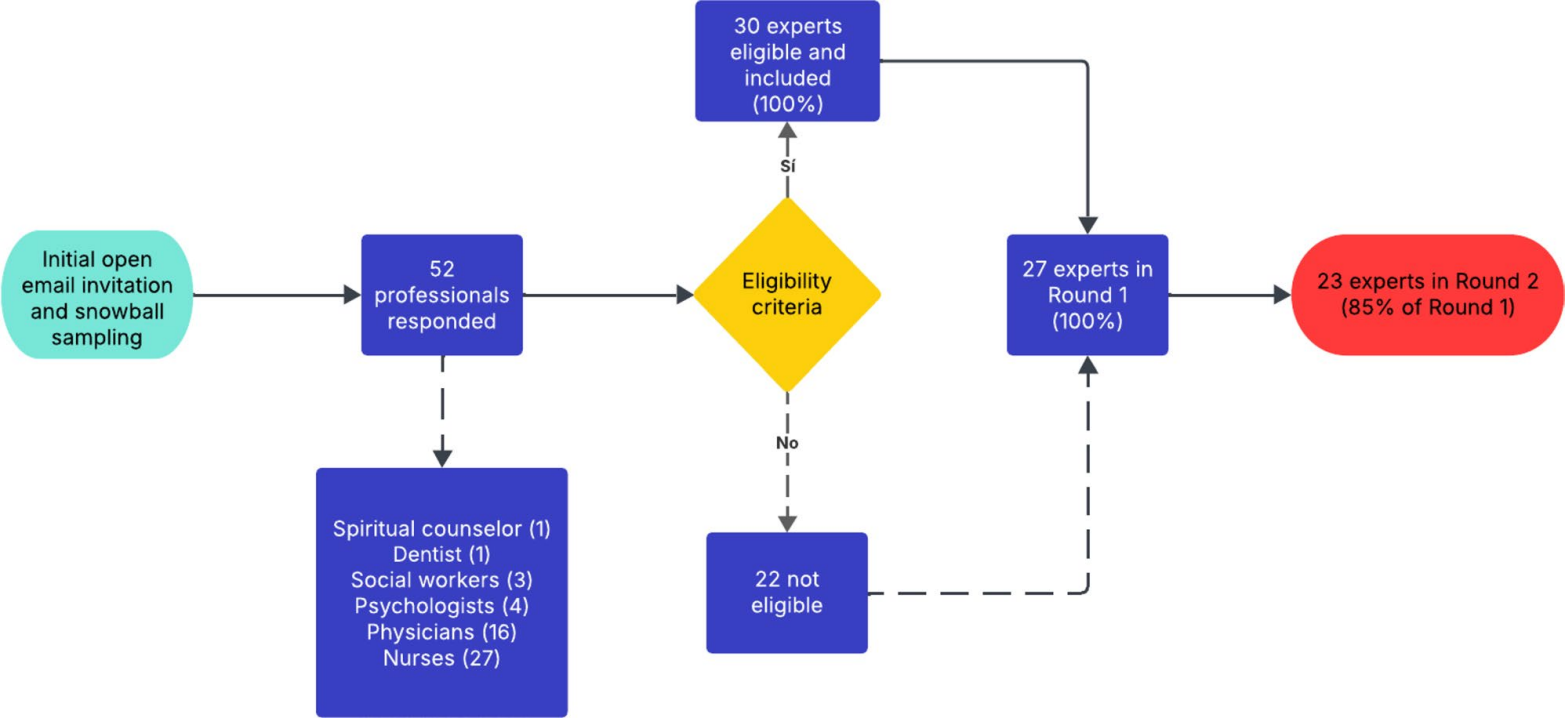
Flowchart of expert participation in the Delphi process.*Legend:* the figure shows the number of professionals who responded to the open invitation and snowball sampling strategy, those who met eligibility criteria, and the number of experts who participated in each Delphi round

Of the 52 professionals who responded to the open invitation and snowball sampling, 30 met eligibility and took part in a separate pre-Delphi to refine the candidate indicators. The Delphi panel then comprised 27 experts in Round 1; 23 completed Round 2 (85% retention; 15% attrition). Between-round analyses were restricted to these 23 experts. Figure 2 shows the participant flow.

The panel included a broad mix of backgrounds: 57% nurses (*n* = 13), 30% physicians (*n* = 7), and 13% other health professionals.

The mean age was 40 years (range 25–62). Regarding experience, 65% (*n* = 15) reported * > *5 years, 26% (*n* = 6) 2–5 years, and 9% (*n* = 2) 1–2 years. Experts represented all seven national nodes that group the 33 Colombian regions [55]. Detailed sociodemographic and expertise information is provided in Table 2.

**Table 2:** Sociodemographic and professional characteristics of the expert panel (*n* = 23)

Area of Experience	Age	Profession	Education Level	Region	Years of Exp.	Field of Expertise
PHC, PH	40	Nurse	PhD	Bogotá	* > *5	Teaching, Research, Community Work
	33		Master’s			Teaching, Community Work
	48		Master’s			Teaching, Administration, Community Work
PC	28	Social Worker	Undergraduate	Bogotá	2–5	Research, Community Work
	40	Physician	Master’s		* > *5	Teaching, Clinical, Research
	56					Clinical, Administration, Project Management
	38	Spiritual Counselor				Clinical, Research, Community Work
	46	Nurse	PhD	Center		Teaching, Research
	41		Master’s	Orinoquia	1–2	Pediatric Care
	51		Specialist	Bogotá	* > *5	
	45		Master’s	Center		Teaching, Clinical, Research
PC, PHC	44	Nurse	Master’s	Pacific	* > *5	Teaching, Clinical, Research, Project Management, Community Work
	44			Bogotá	* > *5	Teaching, Pediatric Clinical, Administration
	36				2–5	Teaching, Clinical, Research, Community Work
PC, PHC, PH	42	Nurse	Master’s	Caribbean	* > *5	Teaching, Administration, Research, Project Management, Community Work
	35			Northeast	1–2	Teaching, Clinical, Research, Community Work, Home-based Palliative Care
	34	Physician		Bogotá	* > *5	Project Management
	30				2–5	Clinical Care
	43			Orinoquia	* > *5	Teaching, Administration, Research
PC, PH	30	Physician	Specialist	Bogotá	2–5	Clinical, Research
	39	Nurse	PhD		2–5	Teaching, Research, Project Management
PH	25	Psychologist	Undergraduate	Amazonia	2–5	Teaching, Administration, Community Work
	62	Physician	Specialist	Orinoquia	* > *5	Teaching, Administration

### Pre-Delphi filtering

The pre-Delphi panel (*n* = 30) rated 69 candidate indicators. Based on the a priori criteria and to reduce participant burden in the main Delphi, 35 indicators were retained for Rounds 1–2. For reporting purposes, indicators directly related to PC were organized according to the six domains of the global framework for PC development (health policies, essential medicines, training and education, service provision, research, and empowerment of people and the empowerment of people and communities). The remaining health-system indicators were classified following the WHO–UNICEF PHC measurement framework [12, 13], covering two of the three PHC components: Integrated health services, Multisectoral policy and action. These were positioned mainly at the levels of structures/inputs, service-delivery processes and outcomes/impact. No indicators were included under the Empowered people & communities component or at the structures/inputs level.

### Delphi consensus results

Consensus thresholds were applied as pre-specified: percentage of ratings *≥*7 had to be *≥*70 and IQR *≤*2. The overall Scale-CVI (S-CVI) was 0.86 in Round 1 and 0.88 in Round 2, indicating moderate-to-high agreement with a small gain between rounds. By dimension, mean CVR exceeded the Lawshe critical value (0.39 for *N=23*) in both rounds: Relevance increased from 0.82 (Round 1) to 0.85 (Round 2); Feasibility remained stable at 0.72; and Validity improved from 0.62 to 0.71.

**Fig. 3:**
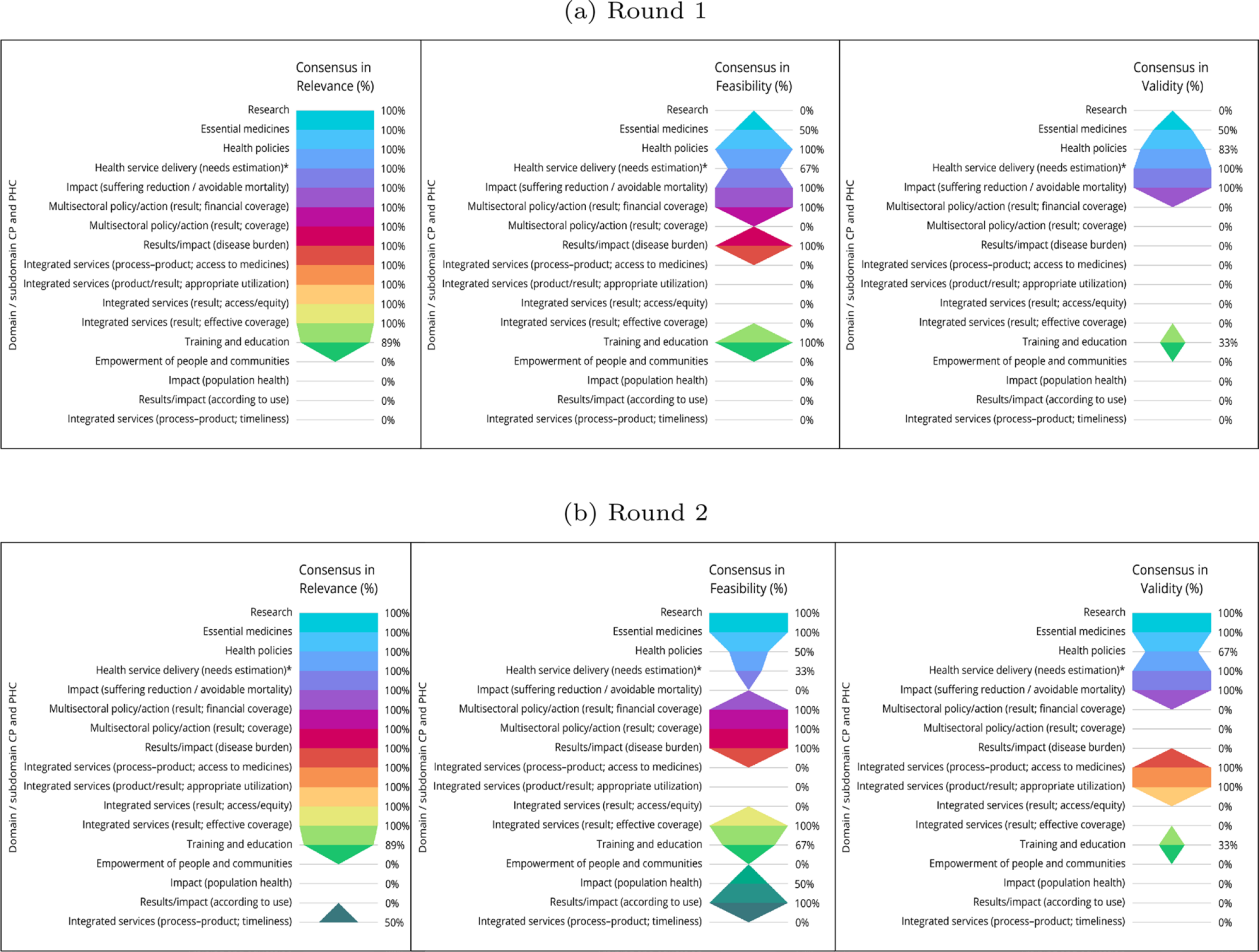
Delphi consensus across PC/PHC domains and dimensions in Round 1 (top) and Round 2 (bottom). *Legend:* comparison of Delphi consensus results across dimensions in Round 1 (top) and Round 2 (bottom). Each panel shows the percentage of indicators within each PC/PHC domain that reached consensus in the three dimensions (relevance, feasibility, and validity)

The share of indicators reaching consensus (by dimension) changed modestly between rounds (Fig. 3).

In Round 1, 80% (*n* = 28/35) achieved consensus for Relevance, 60% (*n* = 21/35) for Feasibility, and 37% (*n* = 13/35) for Validity. In Round 2, Relevance rose to 83% (*n* = 29/35) and Validity to 46% (*n* = 16/35), whereas Feasibility decreased slightly to 54% (*n* = 19/35).

Several indicators consistently showed high agreement and validity across rounds and dimensions (I-CVI *≥*0.83 in both rounds), including: *Existence of a national directory of palliative care services*, *Availability of 24/7 pharmacies for opioid dispensing*, *Existence of licensing standards specific to palliative care services*, *Number of specialized palliative care services per population*, *Continuing education in palliative care for nursing professionals*, and *Medical undergraduate programs with specific palliative care training*. These were judged by experts as relevant, feasible, and valid indicators of PC integration in PHC.

A second group improved from Round 1 to Round 2, particularly on Validity (e.g., *Per capita opioid consumption*, *Palliative care research groups*, and *Nursing undergraduate programs with specific palliative care training*), moving from “relevant/feasible but not yet valid” to acceptable validity in Round 2.

Conversely, some indicators decreased between rounds in at least one dimension (e.g., *Existence of a national palliative care strategy*, *Existence of a national law specific to palliative care*, *Existence of systems for auditing, quality assessment, improvement, or assurance of palliative care services*, *Proportion of home care services offering PC*, *Continuing education in PC for medical professionals*, *Mortality from chronic diseases amenable to PC*), dropping from I-CVI *≥*0.83 in Round 1 to values as low as 0.78 in Round 2 for some dimensions. Detailed classification and CVR/CVI metrics for each indicator are available in Supplementary Tables 2 and 3.

Indicators that did not reach consensus in any dimension included *Presence of professionals affiliated with scientific PC societies*, *Infant mortality rate*, and *Medical consultations per capita rate* (typical I-CVI around 0.57). Non-analytic panel feedback pointed to construct specificity issues. For *Presence of professionals affiliated with scientific PC societies*, several experts noted that affiliation per se does not guarantee service quality or integration and may be uneven across professions; active participation and alignment with PC/PHC practice were viewed as more pertinent. For *Infant mortality rate*, comments suggested restricting to deaths from chronic conditions amenable to PC or adjusting for palliative care access to improve construct validity. For *Medical consultations per capita rate*, experts considered the indicator too general for PC integration and recommended focusing on PC-specific consultations and timeliness within PHC pathways Indicators that did not reach consensus in any dimension included *Presence of professionals affiliated with scientific PC societies*, *Infant mortality rate*, and *Medical consultations per capita rate* (typical I-CVI around 0.57). Non-analytic panel feedback pointed to construct specificity issues. For *Presence of professionals affiliated with scientific PC societies*, several experts noted that affiliation per se does not guarantee service quality or integration and may be uneven across professions; active participation and alignment with PC/PHC practice were viewed as more pertinent. For *Infant mortality rate*, comments suggested restricting to deaths from chronic conditions amenable to PC or adjusting for palliative care access to improve construct validity. For *Medical consultations per capita rate*, experts considered the indicator too general for PC integration and recommended focusing on PC-specific consultations and timeliness within PHC pathways. Illustrative expert comments are provided in Supplementary Table 4 (Spanish originals translated by the authors).

Overall, between-round movement was modest: several indicators consolidated consensus, a smaller set was reappraised downward, and a few remained below threshold. Consistent with the pre-specified stopping rule and the small gain in S-CVI/ (0.86 to 0.88), the process was closed after two rounds to minimize respondent burden.

These results indicate that consensus alone is not sufficient to fully characterize indicator performance, particularly in the presence of heterogeneous expert judgments.

### Profile-based differences in consensus (MAKU analysis)

To explore whether consensus varied according to expert profile, analyses were stratified using the MAKU metric.

#### MAKU metric

The MAKU metric was approximately normally distributed (Shapiro–Wilk *W=0.94*, *p=0.08*). As expected given the heterogeneity of the panel, it allowed meaningful stratification into Strategic and Operational Profile subgroups (Welch’s *t=8.75*, *p < 0.001*, with very large effect sizes: Cohen’s *d≈3.03*, Cliff’s $\delta\approx1.00$). Panel quality remained *moderate* and stable across stages (mean MAKU 0.53 in pre-Delphi, 0.55 in Round 1, 0.53 in Round 2). Sensitivity checks using alternative thresholds (0.35/0.40/0.45) showed *≥*87% consistency in subgroup classification, and robustness tests with *±*10% points in weighting schemes showed *≥*86.7% agreement with the reference classification (Supplementary Figure 10).The MAKU metric was used to stratify the Round 2 panel into Strategic Profile (*n* = 16; MAKU *≥* 0.40) and Operational Profile (*n* = 7; MAKU * < * 0.40) subgroups for analyses.

This stratification revealed that expert profile is a relevant source of variation in consensus outcomes, supporting the need for profile-sensitive analysis in Delphi-based studies.

### Pattern identification and indicator prioritization using SOMs

To further examine patterns in the data and support indicator prioritization, self-organizing maps (SOMs) were applied as a complementary analytical approach.

#### Clustering visualization with SOMs

**Fig. 4:**
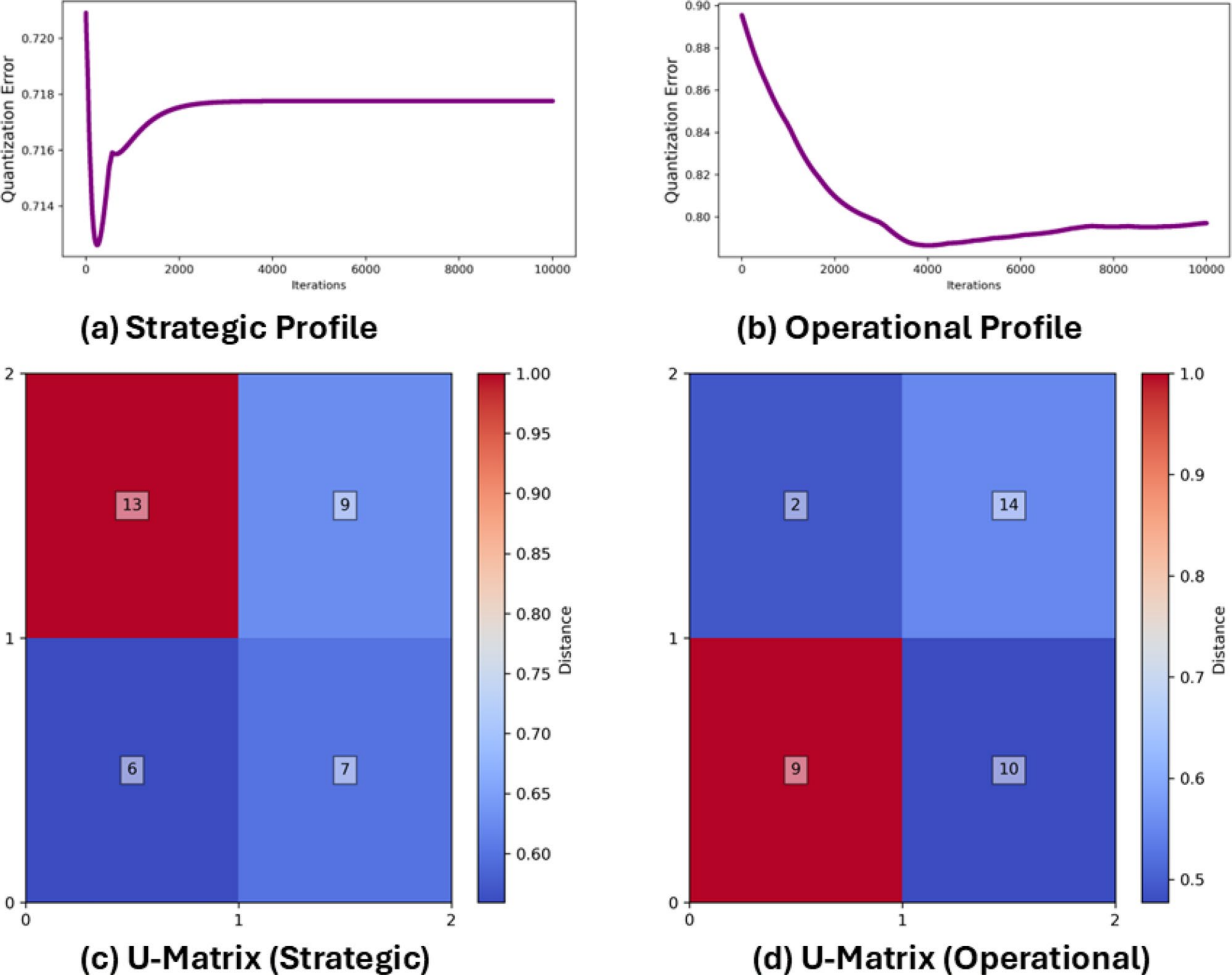
SOM results by expertise profiles. *Legend:* quantization-error curves (top) and U-matrix maps (bottom) for Strategic and Operational profiles

Two 2*×*2 maps were obtained, one per Profile subgroup. The Strategic Profile map converged early and reached a lower final quantization error than the Operational Profile map. Neuron occupancy was more balanced in the Strategic Profile model with 13, 9, 6, and 7 indicators assigned to the four neurons, compared to the Operational Profile model, which had 2, 14, 9, and 10. U-matrix boundaries differed between groups, suggesting that Profile shapes how indicators cluster by “high/low median” and “low/high dispersion” (Fig. 4).

##### Data patterns and pragmatic thresholds

Across both maps, four data patterns were observed by quadrant: (1) high normalized median with low CV_r_ (favorable profile), (2) high median with mid CV_r_ (promising but to be checked for data availability/complementarity), (3) mid median with low CV_r_ (borderline), and (4) low median with high CV_r_ (unfavorable profile, higher dispersion). SOM derived cut points were consistent across groups (Strategic Profile: median *≈*15.7, CV_r_*≈*0.16; Operational Profile: median *≈*16.2 and CV_r_*≈*0.14), aligning with Delphi criteria and helping to visualize which indicators clustered in favorable versus. unfavorable areas.

**Fig. 5:**
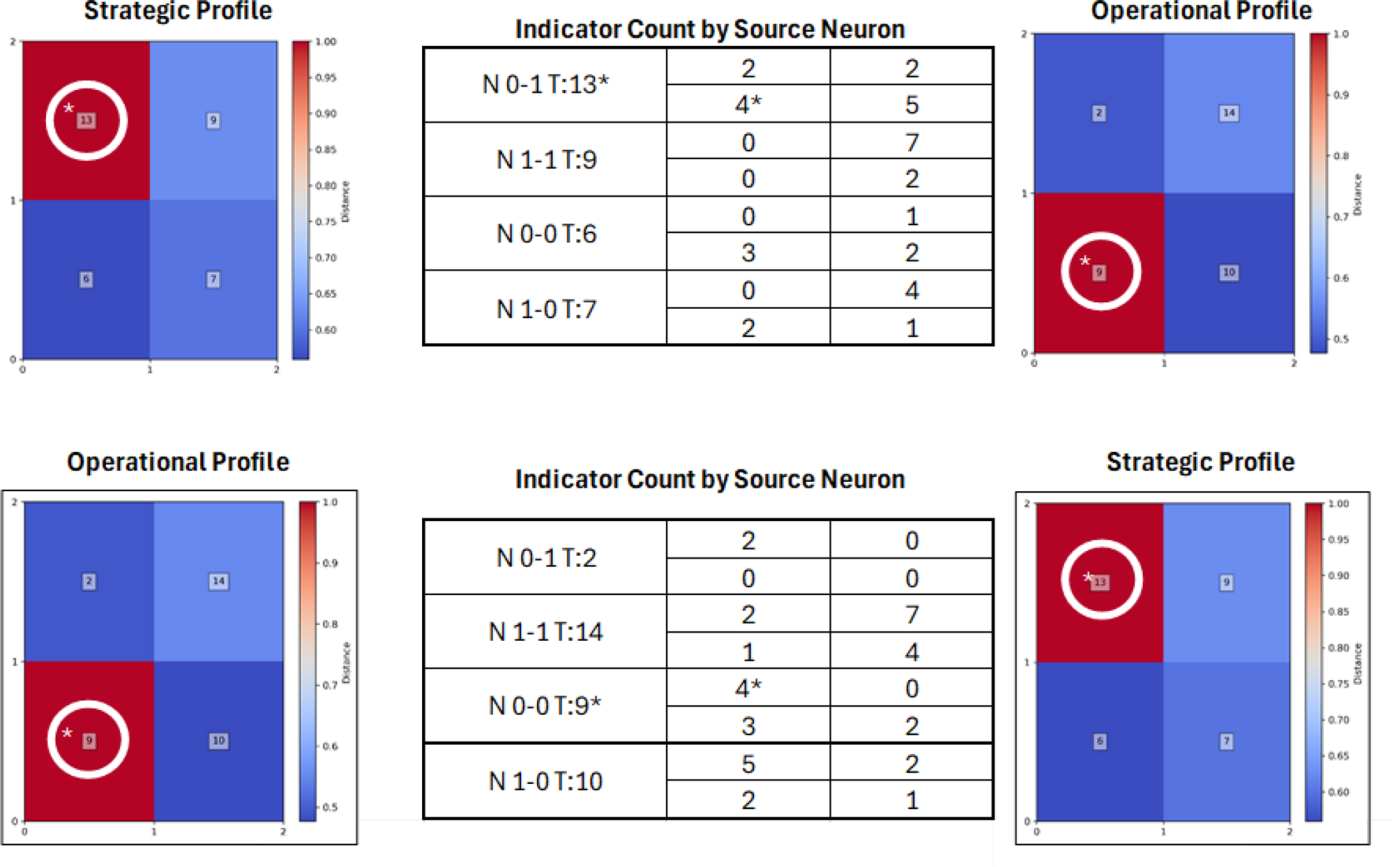
Distribution of indicator assignments across SOM neurons by expert Profile. *Legend:* distribution of indicator assignments between SOM neurons across expert profiles. The tables report, for each source neuron, the number of indicators assigned to each target neuron in the other model. An asterisk (*) marks the dominant neuron in each model (the one with the highest number of assigned indicators). Circles highlight the same neuron in both U-Matrix maps

Using both maps, indicators were compared according to their position in the profile-specific SOMs in order to identify shared and differing prioritization patterns across expert group (Fig. 5). This distinction provides a practical basis for differentiating indicators that are robust across expert profiles from those that require contextual interpretation.

Indicators achieving Delphi consensus were observed across both shared and differing SOM patterns. Full SOM-based indicator assignments, including normalized input values and neuron memberships for both profiles, are provided in Supplementary Tables 5 and 6.

##### Strategic profile SOM

The dominant neuron grouped 13 indicators spanning PC domains and PHC components. Examples include *Availability of 24/7 pharmacies for opioid dispensing*, *Existence of a national palliative care strategy Existence of a national law specific to palliative care*, *Number of specialized palliative care services in the country per population*, *Pediatric palliative care training programs for nursing professionals*, *Nursing and medical undergraduate programs with specific palliative care training*, and *Health insurance coverage rate*. *Proportion of the population with access to basic health services*, Most belonged to *Training and education*, *Health policies*, *Impact (suffering reduction / avoidable mortality)*, and *Integrated services* (effective coverage). A second neuron (6 indicators) contained, among others, *Allocation of funds for palliative care activities in the national health budget by the Ministry of Health*, *Medical faculty with palliative care training*, and *Timely delivery of medications by Health Benefits Plan Administrator (EAPB)*. The least favorable neuron grouped 9 indicators with lower medians and higher dispersion (e.g., items in *Presence of professionals affiliated with scientific palliative care societies*, *Timely assignment of appointments in general medical consultation*, *Medical Consultations per Capita Rate*, and *Infant mortality rate: deaths of children under one year per 1,000 live births* (see Supplementary Table 5).

##### Operational profile SOM

The dominant neuron grouped 9 indicators; five were not present in the Strategic Profile dominant neuron: *Existence of a national directory of palliative care services*, *Allocation of funds for palliative care activities in the national health budget by the Ministry of Health*, *Index of serious health-related suffering*, *Mortality rate from chronic diseases amenable to palliative care*, and *Proportion of hospitalizations due to chronic diseases requiring palliative care*. The least favorable neuron grouped 14 indicators with lower medians (11–14.6) and higher CV_r_ (up to 0.39) (see Supplementary Table 6).

##### Indicator selection and SOM based prioritization

To identify indicators with the most favorable profiles, we focused on the dominant neuron in each SOM map that is, the neuron grouping indicators with the highest median scores and the lowest CV_r_. The Strategic Profile SOM contributed 13 indicators (median: 15.65–17.04; CV_r_: 0.08–0.16), and the Operational Profile SOM contributed 9 indicators (median: 16.19–17.32; CV_r_: 0.14–1.06). After removing duplicates, the final set of 18 indicators was retained see Table 3. Table 3 summarizes the final set of indicators, integrating Delphi consensus results with their conceptual classification and SOM-based prioritization in each expert profile.

**Table 3:** Final set of prioritized indicators and their classification by domain and profile sensitivity

Indicator	PC/PHC domain	Subdomain	Consensus	Operational SOM	Strategic SOM
Availability of 24/7 pharmacies for opioid dispensing	Palliative care development indicators	Essential medicines	Yes	–	X
Existence of a national directory of palliative care services	Palliative care development indicators	Health policies	Yes	X	–
Existence of a national palliative care strategy	Palliative care development indicators	Health policies	Yes	–	X
Allocation of funds for palliative care activities in the national health budget by the Ministry of Health	Palliative care development indicators	Health policies	Yes	X	–
Existence of a national law specific to palliative care	Palliative care development indicators	Health policies	Yes	–	X
Existence of licensing standards specific to palliative care services	Palliative care development indicators	Health policies	Yes	–	X
Index of serious health-related suffering	Palliative care development indicators	Health service delivery (needs estimation)*	Yes	X	–
Number of specialized palliative care services in the country per population	Palliative care development indicators	Health service delivery (needs estimation)*	Yes	X	X
Proportion of home care services offering palliative care	Palliative care development indicators	Health service delivery (needs estimation)*	Yes	X	X
Pediatric palliative care training programs for nursing professionals	Palliative care development indicators	Training and education	Yes	–	X
Pediatric palliative care training programs for medical professionals	Palliative care development indicators	Training and education	Yes	–	X
Nursing undergraduate programs with specific palliative care training	Palliative care development indicators	Training and education	Yes	X	X
Medical undergraduate programs with specific palliative care training	Palliative care development indicators	Training and education	Yes	X	X
Mortality rate from chronic diseases amenable to palliative care	PHC – component (level/subdomain)	Impact (suffering reduction / avoidable mortality)	Yes	X	–
Health insurance coverage rate	PHC – component (level/subdomain)	Multisectoral policy/action (result; financial coverage)	Yes	–	X
Prevalence and mortality rates for chronic diseases (e.g., cancer, heart disease, respiratory disease)	PHC – component (level/subdomain)	Results/impact (disease burden)	Yes	–	X
Proportion of hospitalizations due to chronic diseases requiring palliative care	PHC – component (level/subdomain)	Integrated services (product/result; appropriate utilization)	Yes	X	–
Proportion of the population with access to basic health services	PHC – component (level/subdomain)	Integrated services (result; effective coverage)	Yes	–	X

These indicators showed consistently favorable ratings and low variability within each subgroup, supporting their use in future validation and monitoring efforts, while also illustrating the added value of combining consensus with profile-sensitive and pattern-based analyses (see Supplementary Table 7).

Overall, the SOM analysis complements Delphi consensus by identifying stable and profile-sensitive indicators, providing a more nuanced and transparent basis for indicator prioritization.

## Discussion

### Main findings

This study examines how indicators for measuring the integration of palliative care within primary health care can be more robustly selected when differences in expert profiles are explicitly considered. By combining a modified Delphi approach with profile-based stratification using the MAKU metric and pattern recognition through self-organizing maps (SOMs), we provide a more transparent way to interpret consensus in heterogeneous expert panels.

We prioritised indicators in the dominant neuron of each map (higher median and lower CV_r_). The Strategic Profile map contributed 10 indicators (median 15.65–17.04; CV_r_ 0.08–0.29) and the Operational Profile map contributed 9 (median 15.00–17.32; CV_r_ 0.08–0.21). After removing overlaps, a final set of 18 indicators was retained. Overall agreement was good and slightly higher in Round 2 (S-CVI 0.86 to 0.88). By dimension, mean Lawshe’s CVR increased for Relevance (0.82 to 0.85), remained stable for Feasibility (0.72), and improved for Validity (0.62 to 0.71), suggesting that controlled feedback helped refine judgments in a more abstract domain [29, 56].

Stratifying the panel with the Metric for Assessing Knowledge and Understanding (MAKU) revealed meaningful profile-dependent differences. Two indicators showed the largest contrasts between profiles: *Health insurance coverage rate* (Strategic: median 17.04, CV_r_ 0.16; Operational: median 13.86, CV_r_ 0.30) and *Availability of 24/7 pharmacies for opioid dispensing* (Strategic: median 16.22, CV_r_ 0.11; Operational: median 14.46, CV_r_ 0.15). These profile differences indicate distinct rating patterns. Stratifying by profile prevented masking when pooling the panel and improved the transparency of selection.

A key contribution of this study lies not only in the identification of indicators, but also in showing how consensus-based indicator selection can be strengthened through profile-sensitive and methodologically transparent analysis.

These findings highlight that although both profiles converged in key indicators, the Strategic Profile emphasized policy, workforce training, and educational programs, whereas the Operational Profile gave more weight to service availability, resource allocation, and impact-related indicators. This divergence underscores the value of stratified analysis when exploring expert-derived consensus.

### Interpretation in the context of PC/PHC literature

By focusing on the dominant neuron in each profile-specific map, the selected indicators align with both PC development domains and PHC system components. Experts recognized the value of development indicators (Essential medicines, Health policies, Health service delivery (needs estimation), Training and education), but also emphasized that development does not necessarily imply integration. Hence, indicators from the PHC measurement framework (integrated services, multisectoral action, and empowered people and communities) were viewed as necessary complements to capture system functioning through a PHC lens [12, 13, 57–59].

*Health service delivery (needs estimation)* and *Health policies* featured prominently among the selected items. Added to this, *Essential medicines*, remain central to enabling environments; despite a public-health strategy for PC launched more than a decade ago, large global gaps in access and quality persist [3]. Latin American Country experience also shows that contradictory or unstable primary care policies can jeopardize feasibility of PC integration in PHC, as reported for Brazil during NASF reformulation and funding changes [60]. The interaction between primary care services and the broader PHC approach is mutually reinforcing [10, 61–63].

Training and education were also central. This aligns with the international emphasis on workforce capacity in PC and with Colombia’s policy framework, which prioritizes workforce training and promotes continuing education in PC [9, 15, 17]. A recent thematic synthesis characterized typologies of primary palliative care (PPC) that differ by setting, actors, training requirements, clinical location, services, and financing, underlining the need to embed core PC skills among first-contact providers [64]. Consistent with this, our panel composition, predominantly nurses, echoes calls for undergraduate and in-service education in PC and PHC for nursing and medicine [65], as also emphasized in the Strategic Plan of the Pan American Health Organization 2026–2031 [66]. Importantly, PHC is a whole-of-system approach rather than a single level of care; PPC refers to PC provided by non-specialists at the point of first contact and across community settings, with timely collaboration with specialized PC when needed. PPC is a fundamental pillar of PHC [14].

In the case of the PHC – componente (nivel/subdominio), *Multisectoral policy/action (result; financial coverage)*, *Integrated services (result; effective coverage)* (Proportion of the population with access to basic health services), *Integrated services (product/result; appropriate utilization)* (Proportion of hospitalizations due to chronic diseases requiring palliative care), align with the view that integrated networks, not isolated services, drive continuity across levels, as also noted in the PAHO Strategic Plan on resilient health systems based on primary health care [66, 67]. When patients can move seamlessly among home care, ambulatory care, and hospital care, the system displays the integration that PC requires to be timely and equitable.

Finally, Colombia’s inequity context provides external validity for prioritizing indicators of access, coverage, disease burden, and essential medicines. Although Colombia is classified as level 3b (generalized provision) in global PC development [58], only 12 of 33 subnational regions reach that level; the rest remain in lower categories, mirroring territorial inequities. This heterogeneity was indirectly reflected in our panel composition and supports the case for indicators that are sensitive to geographic and system-level disparities [7, 55, 68].

### Methodological considerations on Delphi

Our results mirror patterns reported in recent methodological overviews of Delphi in the health sciences: relevance often achieves higher agreement than feasibility or validity, and between-round change is modest rather than monotonic [29]. At the same time, long-standing critiques remain: reliance on expert judgment, variable consensus thresholds, selection effects in panel composition, and potential convergence pressure that can suppress dissent [30, 69, 70]. We mitigated these risks through pre-specified thresholds and stopping rules, sensitivity analyses, panel stratification with the MAKU metric, and complementary SOM clustering. For indicators that were not consistently favorable across maps, we recommend phased use with predefined review points rather than immediate exclusion. Notably, CV_r_ was more inclusive than IQR, enabling the retention of indicators with consistent core ratings despite moderate dispersion, particularly in the Operational Profile. This reinforces its utility for decision-making when panel heterogeneity is expected.

### Strengths and limitations

A main strength is the explicit use of the MAKU metric to stratify analyses rather than treating the panel as homogeneous; this sharpened interpretation and supported sensitivity analysis of consensus. The combination of Delphi with SOM-based grouping using median (of the Euclidean norm across experts) and CV_r_ is, to our knowledge, novel in the PC/PHC indicator space. This approach demonstrates that combining structured expert judgment (Delphi), unsupervised learning (SOM), and prior stratification (MAKU) can uncover profile-sensitive differences that would remain hidden in pooled analyses.

Limitations include a modest final panel (*n* = 23) for subgroup analyses; the inherent subjectivity of expert-based consensus; and asynchronous, anonymous procedures that preclude live debate. Our panel was unevenly distributed geographically, with fewer experts from peripheral regions. Finally, some clinicians may be less familiar with machine learning tools such as SOMs, which may limit immediate uptake; this underscores the value of plain-language summaries and capacity-building in data and computational methods within health curriculum.

### Implications for practice and research

For practice and monitoring, indicators prioritized across both profile-specific analyses may be adopted first, whereas indicators showing different SOM positions across profiles may be piloted and reviewed in relation to local priorities and data availability. For research, next steps include: stratifying future Delphi panels by expert characteristics; using complementary clustering to probe robustness; and validating these indicators with real-world data, including, where appropriate, the development of a synthetic indicator.

## Conclusions

The stratification of a modified Delphi panel by expertise profile (MAKU metric) and the use of self-organizing maps showed that consensus patterns are determined by the level of expert experience. A total of 18 indicators were identified to monitor the integration of palliative care into primary health care, demonstrating that a transparent, profile-based selection process enhances the rigor of consensus-based indicator development.

## Electronic supplementary material

Below is the link to the electronic supplementary material.

Supplementary Material 1

Supplementary Material 2

Supplementary Material 3

Supplementary Material 4

Supplementary Material 5

Supplementary Material 6

Supplementary Material 7

Supplementary Material 8

Supplementary Material 9

Supplementary Material 10

## Data Availability

All data and study materials are available in the Supplementary Information files and in the Mendeley Data repository (10.17632/gpgnzkx3bf.3 and 10.17632/7msgr64wz9.1).

## Code availability

All analysis code used in this study is openly available in the Mendeley Data repository under the same (10.17632/gpgnzkx3bf.3 and 10.17632/7msgr64wz9.1).

## Materials availability

All supplementary materials described in this article are also available in the Mendeley Data repository (10.17632/gpgnzkx3bf.3 and 10.17632/7msgr64wz9.1).
