# Supplementary material for: Indicators for palliative care integration in primary health care: how expert profile shapes consensus in a modified Delphi study
Source: BMC Health Serv Res. 2026 Jun 8;26:1119. doi: 10.1186/s12913-026-14781-y (PMC13474756; doi:10.1186/s12913-026-14781-y)
Supplement: Supplementary file 1 [file 12913_2026_14781_MOESM8_ESM.pdf]

**Delphi Round 1**

**Instructions:**

Please evaluate each indicator according to its relevance, feasibility, and validity.
Take a moment to reflect on each criterion before responding.
Your answers will help build a solid consensus for assessing the integration of palliative care into Primary Health Care

**Progress bar**

| Indicator | Formula | Relevance<br>On a scale from 1 (low relevance) to 10 (high relevance), how important is this indicator for assessing the integration of palliative care into PHC? | Feasibility<br>On a scale from 1 (low feasibility) to 10 (high feasibility), how viable is it to measure this indicator in PHC? | Validity<br>On a scale from 1 (low validity) to 10 (high validity), how well does this indicator represent the integration of palliative care into PHC? | Comments: If you believe the indicator should be modified, merged with another, or if a new indicator should be added, please write your comments here |
|---|---|---|---|---|---|
| Access to Health Services in Rural Areas | Proportion of the rural population with access to health services | | | | |
| Accreditation of Official Specialization in Medicine or Palliative Care | Number of institutions offering officially accredited medical specialization in palliative medicine and/or palliative care, by department | | | | |
| Health Insurance Coverage | Proportion of the population with access to any type of health insurance | | | | |
| Opioid Consumption | Morphine-equivalent (ME), excluding methadone, per capita as reported to the INCB (year) | | | | |
| Existence of a National Directory of Palliative Care Services. | Existence of a national directory of palliative care services | | | | |
| Existence of an Up-to-Date National Palliative Care Strategy | Existence of a national and/or departmental palliative care strategy currently in force | | | | |
| Availability of 24/7 Pharmacies for Opioid Dispensing | Number of pharmacies available 24/7 for dispensing state-controlled opioid medications | | | | |
| Allocation of Funds for Palliative Care Activities in the National Health Budget (Ministry of Health) | Allocation of funds for palliative care activities in the national health budget by the Ministry of Health | | | | |
| Palliative Care Research Groups. | Number of research groups in palliative care | | | | |
| Existence of a National Law Specific to Palliative Care | Existence of a national law specifically related to palliative care | | | | |
| Existence of Licensing Standards Specific to Palliative Care Services | Existence of licensing standards specific to the delivery of palliative care services | | | | |
| Number of Specialized Palliative Care Services in the Country per Population | Availability of specialized palliative care services per population. | | | | |
| Timely Assignment of Appointments in General Medical Consultation | Sum of calendar days between the date the patient requests an appointment and the date the appointment is assigned, divided by the total number of general medical consultations | | | | |
| Timely Delivery of Medications by Health Benefits Plan Administrator (EAPB) | Total number of POS medications ordered that are delivered by the pharmacy the first time the affiliate presents the prescription | | | | |
| Population with Access to the General System of Social Security in Health (SGSSS), by Regime | Population with access to the SGSSS by regime (thousands / % share), national total, departments, and area | | | | |
| Prevalence and Mortality Rates for Chronic Diseases | Incidence and mortality rates for diseases such as cancer, heart disease, respiratory disease, and others | | | | |
| Presence of Professionals Affiliated with Scientific Palliative Care Societies | Number of professionals affiliated with national scientific palliative care associations | | | | |
| Medical Faculty with Training in Palliative Care | Number of medical faculty members with training in palliative care | | | | |
| Nursing Faculty with Training in Palliative Care. | Number of nursing faculty members with training in palliative care | | | | |
| Pediatric Palliative Care Training Programs for Medical Professionals | Number of pediatric palliative care training programs for medical professionals | | | | |
| Pediatric Palliative Care Training Programs for Nursing Professionals | Number of pediatric palliative care training programs for nursing professionals | | | | |
| Nursing Undergraduate Programs with Specific Palliative Care Training | Number of undergraduate nursing programs with specific palliative care training | | | | |
| Continuing Education in Palliative Care for Medical Professionals | Number of continuing education programs in palliative care for medical professionals | | | | |
| Continuing Education in Palliative Care for Nursing Professionals | Number of continuing education programs in palliative care for nursing professionals | | | | |
| Medical Undergraduate Programs with Specific Palliative Care Training | Number of undergraduate medical programs with specific palliative care training | | | | |
| Proportion of Hospitalizations due to Chronic Diseases Requiring Palliative Care | Number of hospitalizations related to chronic diseases requiring palliative care | | | | |
| Proportion of the Population with Access to Health Services | Proportion of individuals with access to basic health services, including Primary Health Care | | | | |
| Proportion of Home Care Services Offering Palliative Care | Home care services developed for patients requiring palliative care | | | | |
| Existence of Systems for Auditing, Quality Assessment, Improvement, or Assurance of Palliative Care Services | Existence of systems for auditing, quality assessment, improvement, or assurance of palliative care services | | | | |
| Per Capita Medical Consultation Rate | Average number of medical consultations per inhabitant per year | | | | |
| Mortality Rate from Chronic Diseases Amenable to Palliative Care | Population with chronic diseases amenable to palliative care / Mortality rate | | | | |
| General Mortality Rate | Number of deaths per 1,000 inhabitants in the general population | | | | |
| Infant Mortality Rate | Number of deaths of children under one year per 1,000 live births | | | | |
| In-Hospital Mortality Rate | Number of deaths occurring within hospitals compared with total hospitalizations | | | | |
| Index of Serious Health-Related Suffering in Children Aged 0–14 | Index of suffering from conditions amenable to palliative care in children aged 0–14 / Total deaths in 2019 | | | | |

✕ Missing responses

**Delphi Round 2**

📢 **Instructions:**

**Second Delphi Round – ¡Your input continues to be essential!**

In this round, you will again review the **same indicators** you evaluated previously. You will now see:

🔎 **Group results and initial consensus percentages** (columns D, E, F)

📝 **Qualitative synthesis of comments from Round 1** (column G)

📌 **Your own previous rating for each criterion** (displayed in the corresponding cells)

Please review this information and **record your new evaluation in:**

🎯 Relevance (column L)

⚙️ Feasibility (column M)

📏 Validity (column N)

💬 Optional comment (column O,)

💡 You **may keep your previous rating or adjust** it based on the collective feedback presented in columns L,M,N.

🔁 Only the ratings recorded in this second round (L, M, N), will be considered valid, as we are moving toward the final expert consensus.

🎯 The **goal** of this round is to identify the most appropriate indicators for constructing a synthetic instrument to measure the integration of palliative care into Primary Health Care in Colombia.

🙏 Thank you for your commitment. **We count on you**!

Scroll to the right to record your new evaluation

## RESPONSES AND % AGREEMENT IN DELPHI ROUND 1

| Indicator | Formula | 🎯 Relevance | ⚙️ Feasibility | 📏 Validity | Observations / Comments. Here the analysis of all expert comments from Round 1 is presented |
|---|---|---|---|---|---|
| | | On a scale from 1 (low relevance) to 10 (high relevance), how important is this indicator for assessing the integration of palliative care into PHC?" **Relevance** indicates how important this indicator is for evaluating the integration of palliative care into PHC. An indicator is more relevant if it helps to better understand such integration | On a scale from 1 (low feasibility) to 10 (high feasibility), how feasible is it to measure this indicator in PHC? **Feasibility** assesses whether this indicator can be measured in practice, considering whether data exist, are available, and whether significant resources are needed to obtain them. | On a scale from 1 (low validity) to 10 (high validity), how well does this indicator represent the integration of palliative care into PHC? **Validity** shows whether the indicator clearly reflects what we want to measure. An indicator with high validity faithfully represents the integration of palliative care into primary health care | |
| Access to Health Services in Rural Areas | Proportion of the rural population with access to health services | 🔍 No clear consensus. 63% of experts rated this indicator with a 10, which represents the most frequent score (mode), but does not reflect full consensus among participants. 👉 We invite you to consider this information in your new response. 📝 In the first round, you rated this indicator with a score of: X | 🔍 No clear consensus. 26% of experts rated this indicator with a 10, which represents the most frequent score (mode), but does not reflect full consensus among participants. 👉 We invite you to consider this information in your new response. 📝 In the first round, you rated this indicator with a score of: X | 🔍 No clear consensus. 41% of experts rated this indicator with a 10, which represents the most frequent score (mode), but does not reflect full consensus among participants. 👉 We invite you to consider this information in your new response. 📝 In the first round, you rated this indicator with a score of: X | Comments were received from 10 experts. Experts considered this indicator a useful starting point to explore the integration of PC into PHC, although they highlighted several limitations in its current formulation.<br><br>They indicated that the concept of "access" needs clearer delimitation, incorporating dimensions such as proximity, availability, geographic and economic accessibility. They also noted that the definition of "rural" is ambiguous and may lend itself to local interpretations.<br><br>Several experts highlighted operational barriers to measuring the indicator in rural areas, especially due to lack of data coverage and quality. They also emphasized the importance of trained personnel, multidisciplinary teams, and better alignment with PC. Experts suggest revising the formulation of the indicator and considering the development of sub-indicators that reflect these dimensions. |
| Accreditation of Official Specialization in Medicine or Palliative Care | Number of institutions offering officially accredited medical specialization in palliative medicine and/or palliative care, by department | 🔍 No clear consensus. 37% of experts rated this indicator with a 10, which represents the most frequent score (mode), but does not reflect full consensus among participants. 👉 We invite you to consider this information in your new response. 📝 In the first round, you rated this indicator with a score of: X | 🔍 No clear consensus. 59% of experts rated this indicator with a 10, which represents the most frequent score (mode), but does not reflect full consensus among participants. 👉 We invite you to consider this information in your new response. 📝 In the first round, you rated this indicator with a score of: X | 🔍 No clear consensus. 30% of experts rated this indicator with a 10, which represents the most frequent score (mode), but does not reflect full consensus among participants. 👉 We invite you to consider this information in your new response. 📝 In the first round, you rated this indicator with a score of: X | Comments were received from 8 experts. Experts noted that the term "official accreditation" may be confusing, as it is associated with high-quality standards that do not necessarily reflect the real availability of PC training in the context of PHC.<br><br>Several indicated that this type of accreditation may not be available in all regions, generating a significant territorial gap. Some experts suggested considering undergraduate training as a more accessible alternative to strengthen basic competencies. They also recommended differentiating between accreditation and authorization, and adjusting the indicator's name to improve its understanding and applicability across diverse settings. |
| Health Insurance Coverage | Proportion of the population with access to any type of health insurance | 🔍 No clear consensus. 59% of experts rated this indicator with a 10, which represents the most frequent score (mode), but does not reflect full consensus among participants. 👉 We invite you to consider this information in your new response. 📝 In the first round, you rated this indicator with a score of: X | 🔍 No clear consensus. 44% rated it with a 10, which represents the most frequent score (mode), but does not reflect full consensus among participants. 👉 We invite you to consider this information in your new response. 📝 In the first round, you rated this indicator with a score of: X | 🔍 No clear consensus. 48% rated it with a 10, which represents the most frequent score (mode), but does not reflect full consensus among participants. 👉 We invite you to consider this information in your new response. 📝 In the first round, you rated this indicator with a score of: X | Comments were received from 5 experts. Although experts acknowledged that the indicator can provide an overview of formal access, they noted that affiliation to a health regime does not guarantee effective integration or real access to palliative care.<br><br>They indicated confusion between insurance and effective use of services, especially for populations such as migrants or displaced people. They also questioned the use of the term "health insurance," suggesting replacing it with "Social Security System," and proposed disaggregating information by geographic location to improve sensitivity.<br><br>Experts suggest adjusting the indicator's wording and assessing whether it captures real and effective access to services linked to PC. |
| Opioid Consumption | Morphine-equivalent (ME), excluding methadone, per capita as reported to the INCB (year) | 🔍 No clear consensus. 48% of experts rated this indicator with a 10, which represents the most frequent score (mode), but does not reflect full consensus among participants. 👉 We invite you to consider this information in your new response. 📝 In the first round, you rated this indicator with a score of: X | 🔍 No clear consensus. 26% of experts rated this indicator with a 7, which represents the most frequent score (mode), but does not reflect full consensus among participants. 👉 We invite you to consider this information in your new response. 📝 In the first round, you rated this indicator with a score of: X | 🔍 No clear consensus. 37% of experts rated this indicator with a 10, which represents the most frequent score (mode), but does not reflect full consensus among participants. 👉 We invite you to consider this information in your new response. 📝 In the first round, you rated this indicator with a score of: X | Comments were received from 7 experts. Experts valued the indicator for its potential to reflect access to essential medicines but highlighted important limitations.<br><br>They noted that the indicator focuses on morphine, limiting its usefulness because it excludes other relevant opioids. They also questioned the clarity of the information source (INCB) and the feasibility of obtaining reliable, region-disaggregated data. Some noted that opioid consumption is not always linked to PC, affecting validity.<br><br>Experts suggest reformulating the indicator to include multiple opioids, consider better contextualized data sources, and ensure territorial sensitivity. |
| Existence of a National Directory of Palliative Care Services. | Existence of a national directory of palliative care services | ✅ Strong consensus. 70% of experts rated this indicator with a 10. 👉 We invite you to consider this information in your new response. 📝 In the first round, you rated this indicator with a score of: X | 🔍 No clear consensus. 44% rated it with a 10, which represents the most frequent score (mode), but does not reflect full consensus among participants. 👉 We invite you to consider this information in your new response. 📝 In the first round, you rated this indicator with a score of: X | 🔍 No clear consensus. 48% rated it with a 10, which represents the most frequent score (mode), but does not reflect full consensus among participants. 👉 We invite you to consider this information in your new response. 📝 In the first round, you rated this indicator with a score of: X | Comments were received from 5 experts. Experts valued the indicator's usefulness to improve visibility, articulation, and communication between palliative care services.<br><br>They noted that its existence does not guarantee real access but can facilitate patient orientation, coordination among clinical teams, and dissemination of available services. They also pointed out that the main source (REPS) may not adequately reflect all territories.<br><br>Experts suggest maintaining the indicator but complementing it with measures that evaluate practical use, coverage, and actual capacity to support PC integration into PHC. |
| Existence of an Up-to-Date National Palliative Care Strategy | Existence of a national and/or departmental palliative care strategy currently in force | ✅ Strong consensus. 70% of experts rated this indicator with a 10. 👉 We invite you to consider this information in your new response. 📝 In the first round, you rated this indicator with a score of: X | 🔍 No clear consensus. 26% of experts rated this indicator with an 8, which represents the most frequent score (mode), but does not reflect full consensus among participants. 👉 We invite you to consider this information in your new response. 📝 In the first round, you rated this indicator with a score of: X | 🔍 No clear consensus. 44% of experts rated this indicator with a 10, which represents the most frequent score (mode), but does not reflect full consensus among participants. 👉 We invite you to consider this information in your new response. 📝 In the first round, you rated this indicator with a score of: X | Comments were received from 9 experts. They recognized that the existence of a PC strategy is an important structural element but noted that its presence alone does not guarantee integration into PHC.<br><br>They pointed out that the binary nature (yes/no) is insufficient, as it does not consider implementation level or effective coverage. They questioned alignment with PHC and whether monitoring mechanisms exist.<br><br>Experts suggest reformulating it to incorporate implementation, coverage, PHC alignment, and evaluation mechanisms. |

| | | | | | |
|---|---|---|---|---|---|
| Availability of 24/7 Pharmacies for Opioid Dispensing | Number of pharmacies available 24/7 for dispensing state-controlled opioid medications | 🔍 No clear consensus. 59% of experts rated this indicator with a 10, which represents the most frequent score (mode), but does not reflect full consensus among participants.<br>👉 We invite you to consider this information in your new response.<br>📝 In the first round, you rated this indicator with a score of: X | 🔍 No clear consensus. 33% of experts rated this indicator with an 8, which represents the most frequent score (mode), but does not reflect full consensus among participants.<br>👉 We invite you to consider this information in your new response.<br>📝 In the first round, you rated this indicator with a score of: X | 🔍 No clear consensus. 44% of experts rated this indicator with a 10, which represents the most frequent score (mode), but does not reflect full consensus among participants.<br>👉 We invite you to consider this information in your new response.<br>📝 In the first round, you rated this indicator with a score of: X | Comments were received from 4 experts. They valued the intention of the indicator but noted that its formulation is limited and does not consider whether dispensing actually occurs.<br><br>They indicated the need to consider the entire supply chain and measure real access, not just availability.<br><br>Experts suggest expanding the indicator to evaluate compliance, distribution logistics, and adaptation to the Colombian health system. |
| Allocation of Funds for Palliative Care Activities in the National Health Budget (Ministry of Health) | Allocation of funds for palliative care activities in the national health budget by the Ministry of Health | ✅ Strong consensus. 78% of experts rated this indicator with a 10.<br>👉 We invite you to consider this information in your new response.<br>📝 In the first round, you rated this indicator with a score of: X | 🔍 No clear consensus. 41% of experts rated this indicator with a 10, which represents the most frequent score (mode), but does not reflect full consensus among participants.<br>👉 We invite you to consider this information in your new response.<br>📝 In the first round, you rated this indicator with a score of: X | 🔍 No clear consensus. 52% of experts rated this indicator with a 10, which represents the most frequent score (mode), but does not reflect full consensus among participants.<br>👉 We invite you to consider this information in your new response.<br>📝 In the first round, you rated this indicator with a score of: X | They noted that this may be a sign of institutional commitment, but indicated that its usefulness as an integration indicator depends on also measuring the execution, monitoring, and distribution of these resources.<br><br>They also questioned the clarity of the indicator's formula and its explicit relationship with PHC. They proposed disaggregating the information by type of activity and by region, and incorporating mechanisms that allow assessment of its traceability and actual use.<br><br>They suggest reformulating the indicator to ensure that it measures not only the formal allocation but also the effective use of resources and their impact on the integration of palliative care into PHC. |
| Palliative Care Research Groups. | Number of research groups in palliative care | 🔍 No clear consensus. 52% of experts rated this indicator with a 10, which represents the most frequent score (mode), but does not reflect full consensus among participants.<br>👉 We invite you to consider this information in your new response.<br>📝 In the first round, you rated this indicator with a score of: X | 🔍 No clear consensus. 41% of experts rated this indicator with a 10, which represents the most frequent score (mode), but does not reflect full consensus among participants.<br>👉 We invite you to consider this information in your new response.<br>📝 In the first round, you rated this indicator with a score of: X | 🔍 No clear consensus. 41% of experts rated this indicator with a 10, which represents the most frequent score (mode), but does not reflect full consensus among participants.<br>👉 We invite you to consider this information in your new response.<br>📝 In the first round, you rated this indicator with a score of: X | Comments were received from 4 experts. They noted that the existence of research groups does not necessarily reflect integration with PHC.<br><br>They suggested focusing on scientific production, impact on policies and services, and territorial collaboration.<br><br>Experts recommend reformulating the indicator toward research outcomes and links to PHC needs. |
| Existence of a National Law Specific to Palliative Care | Existence of a national law specifically related to palliative care | ✅ Strong consensus. 93% of experts rated this indicator with a 10.<br>👉 We invite you to consider this information in your new response.<br>📝 In the first round, you rated this indicator with a score of: X | 🔍 No clear consensus. 63% of experts rated this indicator with a 10, which represents the most frequent score (mode), but does not reflect full consensus among participants.<br>👉 We invite you to consider this information in your new response.<br>📝 In the first round, you rated this indicator with a score of: X | 🔍 No clear consensus. 44% of experts rated this indicator with a 10, which represents the most frequent score (mode), but does not reflect full consensus among participants.<br>👉 We invite you to consider this information in your new response.<br>📝 In the first round, you rated this indicator with a score of: X | Comments were received from 7 experts. They recognized the value of a specific law but noted that its existence alone does not guarantee integration.<br><br>They suggested including decrees, resolutions, and other normative instruments, and assessing implementation.<br><br>Experts recommend revising it to evaluate application level, PHC link, and alignment with other policies |
| Existence of Licensing Standards Specific to Palliative Care Services | Existence of licensing standards specific to the delivery of palliative care services | ✅ Strong consensus. 74% of experts rated this indicator with a 10.<br>👉 We invite you to consider this information in your new response.<br>📝 In the first round, you rated this indicator with a score of: X | 🔍 No clear consensus. 52% of experts rated this indicator with a 10, which represents the most frequent score (mode), but does not reflect full consensus among participants.<br>👉 We invite you to consider this information in your new response.<br>📝 In the first round, you rated this indicator with a score of: X | 🔍 No clear consensus. 48% of experts rated this indicator with a 10, which represents the most frequent score (mode), but does not reflect full consensus among participants.<br>👉 We invite you to consider this information in your new response.<br>📝 In the first round, you rated this indicator with a score of: X | Comments were received from 4 experts. The experts considered this indicator relevant, but noted that the current licensing standards are not specifically formulated for palliative care and may represent a barrier rather than a tool for its development.<br><br>They indicated that these standards are usually focused on general medicine, without a clear connection to PHC or the particular characteristics of palliative care. They also proposed adjusting the wording of the indicator to align it with existing legal regulations.<br><br>The experts suggest reformulating this indicator to assess not only its existence, but also its applicability, specificity, and articulation with Primary Health Care |
| Number of Specialized Palliative Care Services in the Country per Population | Availability of specialized palliative care services per population. | ✅ Strong consensus. 70% of experts rated this indicator with a 10.<br>👉 We invite you to consider this information in your new response.<br>📝 In the first round, you rated this indicator with a score of: X | 🔍 Sin consenso claro. El 41% de los expertos calificó este indicador con un 9, which represents the most frequent score (mode), but does not reflect full consensus among participants.<br><br>👉 We invite you to consider this information in your new response.<br>📝 In the first round, you rated this indicator with a score of: X | 🔍 Sin consenso claro. El 41% de los expertos calificó este indicador con un 10, which represents the most frequent score (mode), but does not reflect full consensus among participants.<br><br>👉 We invite you to consider this information in your new response.<br>📝 In the first round, you rated this indicator with a score of: X | Comments were received from 5 experts. The experts highlighted the usefulness of the indicator but pointed out that the concept of "specific services" is ambiguous and requires a clearer definition.<br><br>They suggested distinguishing between modalities such as interconsultation, structured programs, hospital units, among others, and that this distinction should be reflected. They also emphasized the importance of disaggregating information by region and explicitly linking it to the PHC context.<br><br>The experts suggest clarifying the focus of the indicator and considering a clearer typology of the services included, as well as their geographic distribution. |
| Timely Assignment of Appointments in General Medical Consultation | Sum of calendar days between the date the patient requests an appointment and the date the appointment is assigned, divided by the total number of general medical consultations | 🔍 No clear consensus. 52% of experts rated this indicator with a 10, which represents the most frequent score (mode), but does not reflect full consensus among participants.<br>👉 We invite you to consider this information in your new response.<br>📝 In the first round, you rated this indicator with a score of: X | 🔍 No clear consensus. 44% of experts rated this indicator with a 10, which represents the most frequent score (mode), but does not reflect full consensus among participants.<br>👉 We invite you to consider this information in your new response.<br>📝 In the first round, you rated this indicator with a score of: X | 🔍 No clear consensus. 30% of experts rated this indicator with a 10, which represents the most frequent score (mode), but does not reflect full consensus among participants.<br>👉 We invite you to consider this information in your new response.<br>📝 In the first round, you rated this indicator with a score of: X | Comments were received from 5 experts. The experts considered that this indicator, as currently formulated, reflects access to general medical consultation but does not necessarily measure the integration of palliative care into PHC.<br><br>They indicated that the metric is already included in regulatory systems and that its focus appears to be more related to general or specialized care. They proposed developing a more specific indicator on timeliness of palliative care attention. |
| Timely Delivery of Medications by Health Benefits Plan Administrator (EAPB) | Total number of POS medications ordered that are delivered by the pharmacy the first time the affiliate presents the prescription | 🔍 No clear consensus. 67% of experts rated this indicator with a 10, which represents the most frequent score (mode), but does not reflect full consensus among participants.<br>👉 We invite you to consider this information in your new response.<br>📝 In the first round, you rated this indicator with a score of: X | 🔍 No clear consensus. 48% of experts rated this indicator with a 10, which represents the most frequent score (mode), but does not reflect full consensus among participants.<br>👉 We invite you to consider this information in your new response.<br>📝 In the first round, you rated this indicator with a score of: X | 🔍 No clear consensus. 52% of experts rated this indicator with a 10, which represents the most frequent score (mode), but does not reflect full consensus among participants.<br>👉 We invite you to consider this information in your new response.<br>📝 In the first round, you rated this indicator with a score of: X | Comments were received from 5 experts. The experts noted that this indicator is operationally relevant, but its current formulation is broad and not specific to palliative care.<br><br>They indicated that it is unclear whether it includes all relevant medications, devices, and supplies, and whether it considers POS, PBS, or MIPRES. They also noted that its applicability is limited to the Colombian system, which restricts generalization.<br><br>The experts suggest reformulating the indicator to focus on key palliative care medications and devices and ensure a clear definition of its scope. |
| Population with Access to the General System of Social Security in Health (SGSSS), by Regime | Population with access to the SGSSS by regime (thousands / % share), national total, departments, and area | 🔍 No clear consensus. 59% of experts rated this indicator with a 10, which represents the most frequent score (mode), but does not reflect full consensus among participants.<br>👉 We invite you to consider this information in your new response.<br>📝 In the first round, you rated this indicator with a score of: X | 🔍 No clear consensus. 52% of experts rated this indicator with a 10, which represents the most frequent score (mode), but does not reflect full consensus among participants.<br>👉 We invite you to consider this information in your new response.<br>📝 In the first round, you rated this indicator with a score of: X | 🔍 No clear consensus. 44% of experts rated this indicator with a 10, which represents the most frequent score (mode), but does not reflect full consensus among participants.<br>👉 We invite you<br>📝 In the first round, you rated this indicator with a score of: X | Comments were received from 5 experts. The experts considered the indicator a useful source of context, but noted that it does not reflect palliative care integration in PHC.<br><br>They indicated that affiliation does not imply effective care or coverage of palliative services. They also recommended considering non-affiliated populations and the barriers to their inclusion. The usefulness of the term "with insurance coverage" was questioned due to its ambiguity.<br><br>The experts suggest reviewing the formulation and considering additional variables to more precisely capture the link with palliative care. |
| Prevalence and Mortality Rates for Chronic Diseases | Incidence and mortality rates for diseases such as cancer, heart disease, respiratory disease, and others | 🔍 No clear consensus. 67% of experts rated this indicator with a 10, which represents the most frequent score (mode), but does not reflect full consensus among participants.<br>👉 We invite you to consider this information in your new response.<br>📝 In the first round, you rated this indicator with a score of: X | 🔍 No clear consensus. 59% of experts rated this indicator with a 10, which represents the most frequent score (mode), but does not reflect full consensus among participants.<br>👉 We invite you to consider this information in your new response.<br>📝 In the first round, you rated this indicator with a score of: X | 🔍 No clear consensus. 52% of experts rated this indicator with a 10, which represents the most frequent score (mode), but does not reflect full consensus among participants.<br>👉 We invite you to consider this information in your new response.<br>📝 In the first round, you rated this indicator with a score of: X | Comments were received from 4 experts. The experts recognized that this indicator allows an approximation to palliative care needs but stated that it does not directly reflect PHC integration.<br><br>They highlighted the need to distinguish between prevalence, incidence, and mortality, and proposed expanding the focus to include degenerative or life-threatening diseases. Concerns were also raised about data validity and practical applicability.<br><br>The experts suggest adjusting the indicator to improve its conceptual precision and alignment with the objective of this research. |

| | | | | | |
|---|---|---|---|---|---|
| Presence of Professionals Affiliated with Scientific Palliative Care Societies | Number of professionals affiliated with national scientific palliative care associations | 🔍 No clear consensus. 41% of experts rated this indicator with an 8, which represents the most frequent score (mode), but does not reflect full consensus among participants.<br>👉 We invite you to consider this information in your new response.<br>📝 In the first round, you rated this indicator with a score of: X | 🔍 No clear consensus. 44% of experts rated this indicator with a 10, which represents the most frequent score (mode), but does not reflect full consensus among participants.<br>👉 We invite you to consider this information in your new response.<br>📝 In the first round, you rated this indicator with a score of: X | 🔍 No clear consensus. 26% of experts rated this indicator with a 7, which represents the most frequent score (mode), but does not reflect full consensus among participants.<br>👉 We invite you to consider this information in your new response.<br>📝 In the first round, you rated this indicator with a score of: X | Comments were received from 4 experts. The experts noted that affiliation with scientific societies does not guarantee integration with PHC nor real impact on care.<br><br>They indicated that this indicator does not directly reflect service provision and may vary depending on the type of professional. They also mentioned that promotion of membership is low.<br><br>The experts suggest revising the indicator to focus on active participation and commitment to clinical practices aligned with palliative care and PHC. |
| Medical Faculty with Training in Palliative Care | Number of medical faculty members with training in palliative care | 🔍 No clear consensus. 59% of experts rated this indicator with a 10, which represents the most frequent score (mode), but does not reflect full consensus among participants.<br>👉 We invite you to consider this information in your new response.<br>📝 In the first round, you rated this indicator with a score of: X | 🔍 No clear consensus. 44% of experts rated this indicator with a 10, which represents the most frequent score (mode), but does not reflect full consensus among participants.<br>👉 We invite you to consider this information in your new response.<br>📝 In the first round, you rated this indicator with a score of: X | 🔍 No clear consensus. 41% of experts rated this indicator with a 10, which represents the most frequent score (mode), but does not reflect full consensus among participants.<br>👉 We invite you to consider this information in your new response.<br>📝 In the first round, you rated this indicator with a score of: X | Comments were received from 3 experts. The experts valued faculty training but noted that having trained professors does not guarantee palliative care integration into PHC.<br><br>They proposed orienting the indicator toward curricular content, PHC focus, and learning outcomes. They also noted that the term "palliative care training" is broad and must be specified.<br><br>The experts suggest reformulating the indicator to evaluate curricular integration of palliative care in medical programs with emphasis on PHC. |
| Nursing Faculty with Training in Palliative Care. | Number of nursing faculty members with training in palliative care | 🔍 No clear consensus. 59% of experts rated this indicator with a 10, which represents the most frequent score (mode), but does not reflect full consensus among participants.<br>👉 We invite you to consider this information in your new response.<br>📝 In the first round, you rated this indicator with a score of: X | 🔍 No clear consensus. 48% of experts rated this indicator with a 10, which represents the most frequent score (mode), but does not reflect full consensus among participants.<br>👉 We invite you to consider this information in your new response.<br>📝 In the first round, you rated this indicator with a score of: X | 🔍 No clear consensus. 41% of experts rated this indicator with a 10, which represents the most frequent score (mode), but does not reflect full consensus among participants.<br>👉 We invite you to consider this information in your new response.<br>📝 In the first round, you rated this indicator with a score of: X | Comments were received from 3 experts. The experts considered the indicator useful as a structural measure but noted that its usefulness for evaluating integration is limited.<br><br>They indicated that it is unclear whether the indicator considers only faculty or also clinical professionals. They also recommended explicitly linking palliative care training with PHC.<br><br>The experts suggest strengthening the indicator by incorporating dimensions such as professional impact, pedagogical approach, and PHC content. |
| Pediatric Palliative Care Training Programs for Medical Professionals | Number of pediatric palliative care training programs for medical professionals | 🔍 No clear consensus. 59% of experts rated this indicator with a 10, which represents the most frequent score (mode), but does not reflect full consensus among participants.<br>👉 We invite you to consider this information in your new response.<br>📝 In the first round, you rated this indicator with a score of: X | 🔍 No clear consensus. 52% of experts rated this indicator with a 10, which represents the most frequent score (mode), but does not reflect full consensus among participants.<br>👉 We invite you to consider this information in your new response.<br>📝 In the first round, you rated this indicator with a score of: X | 🔍 No clear consensus. 41% of experts rated this indicator with a 10, which represents the most frequent score (mode), but does not reflect full consensus among participants.<br>👉 We invite you to consider this information in your new response.<br>📝 In the first round, you rated this indicator with a score of: X | Comments were received from 3 experts. The experts valued the indicator for its focus on specialized training but noted that its existence does not guarantee integration with PHC.<br><br>They suggested that it would be useful to know how many graduates work with a PHC approach in pediatric palliative care. They also recommended assessing training quality, hours dedicated, and clinical practice.<br><br>The experts suggest reformulating the indicator to include aspects related to professional impact, curricular focus, and PHC articulation. |
| Pediatric Palliative Care Training Programs for Nursing Professionals | Number of pediatric palliative care training programs for nursing professionals | 🔍 No clear consensus. 59% of experts rated this indicator with a 10, which represents the most frequent score (mode), but does not reflect full consensus among participants.<br>👉 We invite you to consider this information in your new response.<br>📝 In the first round, you rated this indicator with a score of: X | 🔍 No clear consensus. 52% of experts rated this indicator with a 10, which represents the most frequent score (mode), but does not reflect full consensus among participants.<br>👉 We invite you to consider this information in your new response.<br>📝 In the first round, you rated this indicator with a score of: X | 🔍 No clear consensus. 41% of experts rated this indicator with a 10, which represents the most frequent score (mode), but does not reflect full consensus among participants.<br>👉 We invite you to consider this information in your new response.<br>📝 In the first round, you rated this indicator with a score of: X | Comments were received from 3 experts. The experts positively valued the existence of pediatric palliative care training programs for nursing but noted the need to ensure their orientation toward PHC.<br><br>They indicated that many programs combine adult and pediatric PC, which could dilute specificity. They also recommended reviewing curricular content and the care approach and explicitly linking training with PHC principles.<br><br>The experts suggest maintaining this indicator with adjustments that ensure its exclusive pediatric focus and PHC connection. |
| Nursing Undergraduate Programs with Specific Palliative Care Training | Number of undergraduate nursing programs with specific palliative care training | ✅ Strong consensus. 74% of experts rated this indicator with a 10.<br>👉 We invite you to consider this information in your new response.<br>📝 In the first round, you rated this indicator with a score of: X | 🔍 No clear consensus. 52% of experts rated this indicator with a 10, which represents the most frequent score (mode), but does not reflect full consensus among participants.<br>👉 We invite you to consider this information in your new response.<br>📝 In the first round, you rated this indicator with a score of: X | 🔍 No clear consensus. 52% of experts rated this indicator with a 10, which represents the most frequent score (mode), but does not reflect full consensus among participants.<br>👉 We invite you to consider this information in your new response.<br>📝 In the first round, you rated this indicator with a score of: X | Comments were received from 3 experts. The experts considered this a valuable structural indicator but noted that it should be strengthened with criteria that assess its impact on professional practice.<br><br>They highlighted the importance of knowing how many graduates work in PHC with a palliative care approach, as well as reviewing course load, curricular content, and clinical practice. They also proposed explicitly including PHC components in training.<br><br>The experts suggest maintaining the indicator while incorporating criteria that ensure quality, applicability, and PHC focus. |
| Continuing Education in Palliative Care for Medical Professionals | Number of continuing education programs in palliative care for medical professionals | 🔍 No clear consensus. 67% of experts rated this indicator with a 10, which represents the most frequent score (mode), but does not reflect full consensus among participants.<br>👉 We invite you to consider this information in your new response.<br>📝 In the first round, you rated this indicator with a score of: X | 🔍 No clear consensus. 48% of experts rated this indicator with a 10, which represents the most frequent score (mode), but does not reflect full consensus among participants.<br>👉 We invite you to consider this information in your new response.<br>📝 In the first round, you rated this indicator with a score of: X | 🔍 No clear consensus. 41% of experts rated this indicator with a 10, which represents the most frequent score (mode), but does not reflect full consensus among participants.<br>👉 We invite you to consider this information in your new response.<br>📝 In the first round, you rated this indicator with a score of: X | One comment was received. The expert recognized the importance of continuing education but noted that there is little evidence of its real impact on palliative care integration into PHC.<br><br>They indicated that the indicator, as currently formulated, does not allow assessment of whether these programs generate concrete changes in clinical practice.<br><br>The expert suggests reformulating the indicator to include tools that evaluate outcomes or transformations associated with continuing education. |
| Continuing Education in Palliative Care for Nursing Professionals | Number of continuing education programs in palliative care for nursing professionals | 🔍 No clear consensus. 67% of experts rated this indicator with a 10, which represents the most frequent score (mode), but does not reflect full consensus among participants.<br>👉 We invite you to consider this information in your new response.<br>📝 In the first round, you rated this indicator with a score of: X | 🔍 No clear consensus. 48% of experts rated this indicator with a 10, which represents the most frequent score (mode), but does not reflect full consensus among participants.<br>👉 We invite you to consider this information in your new response.<br>📝 In the first round, you rated this indicator with a score of: X | 🔍 No clear consensus. 41% of experts rated this indicator with a 10, which represents the most frequent score (mode), but does not reflect full consensus among participants.<br>👉 We invite you to consider this information in your new response.<br>📝 In the first round, you rated this indicator with a score of: X | One comment was received. The expert valued the existence of continuing education programs as a positive initiative but noted that the indicator, in its current form, does not allow assessment of PC–PHC integration.<br><br>They indicated that evidence of impact on professional practice is lacking and that mechanisms to measure real effects are needed.<br><br>The expert suggests maintaining the indicator with adjustments that link it to concrete results in the PHC context. |
| Medical Undergraduate Programs with Specific Palliative Care Training | Number of undergraduate medical programs with specific palliative care training | ✅ Strong consensus. 70% of experts rated this indicator with a 10.<br>👉 We invite you to consider this information in your new response.<br>📝 In the first round, you rated this indicator with a score of: X | 🔍 No clear consensus. 52% of experts rated this indicator with a 10, which represents the most frequent score (mode), but does not reflect full consensus among participants.<br>👉 We invite you to consider this information in your new response.<br>📝 In the first round, you rated this indicator with a score of: X | 🔍 No clear consensus. 44% of experts rated this indicator with a 10, which represents the most frequent score (mode), but does not reflect full consensus among participants.<br>👉 We invite you to consider this information in your new response.<br>📝 In the first round, you rated this indicator with a score of: X | One comment was received. The expert valued the existence of educational programs in palliative care but noted that their link to PHC is not clear.<br><br>They indicated that some programs may include PC as a subject without focusing on PHC, which limits usefulness.<br><br>The expert suggests reformulating the indicator to include assessment of curricular content and its explicit link to PHC. |
| Proportion of Hospitalizations due to Chronic Diseases Requiring Palliative Care | Number of hospitalizations related to chronic diseases requiring palliative care | ✅ Strong consensus. 74% of experts rated this indicator with a 10.<br>👉 We invite you to consider this information in your new response.<br>📝 In the first round, you rated this indicator with a score of: X | 🔍 No clear consensus. 44% of experts rated this indicator with a 10, which represents the most frequent score (mode), but does not reflect full consensus among participants.<br>👉 We invite you to consider this information in your new response.<br>📝 In the first round, you rated this indicator with a score of: X | 🔍 No clear consensus. 48% of experts rated this indicator with a 10, which represents the most frequent score (mode), but does not reflect full consensus among participants.<br>👉 We invite you to consider this information in your new response.<br>📝 In the first round, you rated this indicator with a score of: X | Comments were received from 3 experts. The experts recognized the potential of the indicator to reflect palliative care needs but noted that, as formulated, it presents important ambiguities.<br><br>They indicated that causes of hospitalization for chronic diseases can vary and are not always related to palliative care needs. They also proposed disaggregating data by territory and linking it more clearly to diseases plausible for palliative care.<br><br>The experts suggest adjusting the indicator formulation to strengthen its validity as an indirect approximation of integration. |
| Proportion of the Population with Access to Health Services | Proportion of individuals with access to basic health services, including Primary Health Care | 🔍 No clear consensus. 52% of experts rated this indicator with a 10, which represents the most frequent score (mode), but does not reflect full consensus among participants.<br>👉 We invite you to consider this information in your new response.<br>📝 In the first round, you rated this indicator with a score of: X | 🔍 No clear consensus. 48% of experts rated this indicator with a 10, which represents the most frequent score (mode), but does not reflect full consensus among participants.<br>👉 We invite you to consider this information in your new response.<br>📝 In the first round, you rated this indicator with a score of: X | 🔍 No clear consensus. 48% of experts rated this indicator with a 10, which represents the most frequent score (mode), but does not reflect full consensus among participants.<br>👉 We invite you to consider this information in your new response.<br>📝 In the first round, you rated this indicator with a score of: X | Comments were received from 4 experts. The experts valued this indicator as relevant and feasible, highlighting its usefulness as a starting point to characterize access to health services.<br><br>However, they noted that its current formulation is too general and does not specifically reflect palliative care integration. They indicated that it is important to specify which services are included and whether they correspond to the first level with capacity for PC.<br><br>The experts suggest maintaining the indicator while adjusting it to improve specificity and focus on services linked to palliative care in PHC. |

| | | | | | |
|---|---|---|---|---|---|
| Proportion of Home Care Services Offering Palliative Care | Home care services developed for patients requiring palliative care | ✅ Strong consensus. 81% of experts rated this indicator with a 10.<br>👉 We invite you to consider this information in your new response.<br>📝 In the first round, you rated this indicator with a score of: X | 🔍 No clear consensus. 59% of experts rated this indicator with a 10, which represents the most frequent score (mode), but does not reflect full consensus among participants.<br>👉 We invite you to consider this information in your new response.<br>📝 In the first round, you rated this indicator with a score of: X | 🔍 No clear consensus. 56% of experts rated this indicator with a 10, which represents the most frequent score (mode), but does not reflect full consensus among participants.<br>👉 We invite you to consider this information in your new response.<br>📝 In the first round, you rated this indicator with a score of: X | Comments were received from 2 experts. The experts highly valued this indicator, considering it a clear representation of palliative care integration in community and home settings.<br><br>They noted the importance of monitoring not only the existence of services but also their actual provision. They also indicated that this indicator aligns closely with PHC principles.<br><br>The experts suggest maintaining the indicator and complementing it with tools that assess effective service delivery. |
| Existence of Systems for Auditing, Quality Assessment, Improvement, or Assurance of Palliative Care Services | Existence of systems for auditing, quality assessment, improvement, or assurance of palliative care services | 🔍 No clear consensus. 59% of experts rated this indicator with a 10, which represents the most frequent score (mode), but does not reflect full consensus among participants.<br>👉 We invite you to consider this information in your new response.<br>📝 In the first round, you rated this indicator with a score of: X | 🔍 No clear consensus. 33% of experts rated this indicator with a 10, which represents the most frequent score (mode), but does not reflect full consensus among participants.<br>👉 We invite you to consider this information in your new response.<br>📝 In the first round, you rated this indicator with a score of: X | 🔍 No clear consensus. 48% of experts rated this indicator with a 10, which represents the most frequent score (mode), but does not reflect full consensus among participants.<br>👉 We invite you to consider this information in your new response.<br>📝 In the first round, you rated this indicator with a score of: X | Comments were received from 4 experts. The experts considered this indicator relevant but noted that it may overlap with already existing general regulatory systems.<br><br>They highlighted the need to better define its scope, specify the entities involved, and clarify audit criteria focused on PC and PHC. They also suggested differentiating it from other regulatory frameworks.<br><br>The experts suggest adjusting the indicator to improve operability, specificity, and differentiation within the health quality system. |
| Per Capita Medical Consultation Rate | Average number of medical consultations per inhabitant per year | 🔍 No clear consensus. 41% of experts rated this indicator with a 10, which represents the most frequent score (mode), but does not reflect full consensus among participants.<br>👉 We invite you to consider this information in your new response.<br>📝 In the first round, you rated this indicator with a score of: X | 🔍 No clear consensus. 41% of experts rated this indicator with a 10, which represents the most frequent score (mode), but does not reflect full consensus among participants.<br>👉 We invite you to consider this information in your new response.<br>📝 In the first round, you rated this indicator with a score of: X | 🔍 No clear consensus. 30% of experts rated this indicator with a 10, which represents the most frequent score (mode), but does not reflect full consensus among participants.<br>👉 We invite you to consider this information in your new response.<br>📝 In the first round, you rated this indicator with a score of: X | Comments were received from 5 experts. The experts noted that this indicator reflects general access to medical services but does not assess palliative care integration in PHC.<br><br>They indicated that it is too broad and already available in official sources. They recommended reformulating it to focus on consultations specifically related to palliative care.<br><br>The experts suggest developing an alternative indicator that captures access to medical services with a palliative focus at the first level of care. |
| Mortality Rate from Chronic Diseases Amenable to Palliative Care | Population with chronic diseases amenable to palliative care / Mortality rate | ✅ Strong consensus. 74% of experts rated this indicator with a 10.<br>👉 We invite you to consider this information in your new response.<br>📝 In the first round, you rated this indicator with a score of: X | 🔍 No clear consensus. 52% of experts rated this indicator with a 10, which represents the most frequent score (mode), but does not reflect full consensus among participants.<br>👉 We invite you to consider this information in your new response.<br>📝 In the first round, you rated this indicator with a score of: X | 🔍 No clear consensus. 56% of experts rated this indicator with a 10, which represents the most frequent score (mode), but does not reflect full consensus among participants.<br>👉 We invite you to consider this information in your new response.<br>📝 In the first round, you rated this indicator with a score of: X | Comments were received from 3 experts. The experts valued this indicator for estimating potential palliative care demand but noted some technical limitations.<br><br>They indicated that data quality may vary by territory and that the formula requires adjustments to improve precision.<br><br>The experts suggest maintaining the indicator while strengthening its technical basis and ensuring geographic disaggregation. |
| General Mortality Rate | Number of deaths per 1,000 inhabitants in the general population | 🔍 No clear consensus. 44% of experts rated this indicator with a 10, which represents the most frequent score (mode), but does not reflect full consensus among participants.<br>👉 We invite you to consider this information in your new response.<br>📝 In the first round, you rated this indicator with a score of: X | 🔍 No clear consensus. 59% of experts rated this indicator with a 10, which represents the most frequent score (mode), but does not reflect full consensus among participants.<br>👉 We invite you to consider this information in your new response.<br>📝 In the first round, you rated this indicator with a score of: X" | 🔍 No clear consensus. 33% of experts rated this indicator with a 10, which represents the most frequent score (mode), but does not reflect full consensus among participants.<br>👉 We invite you to consider this information in your new response.<br>📝 In the first round, you rated this indicator with a score of: X | Comments were received from 5 experts. The experts considered this indicator provides contextual information but is not specific for assessing PC–PHC integration.<br><br>They noted that it is already available in official sources and overlaps with more targeted indicators of chronic or PC-related conditions.<br><br>The experts suggest not prioritizing this indicator due to its generality and low alignment with the study objective. |
| Infant Mortality Rate | Number of deaths of children under one year per 1,000 live births | 🔍 No clear consensus. 48% of experts rated this indicator with a 10, which represents the most frequent score (mode), but does not reflect full consensus among participants.<br>👉 We invite you to consider this information in your new response.<br>📝 In the first round, you rated this indicator with a score of: X | 🔍 No clear consensus. 52% of experts rated this indicator with a 10, which represents the most frequent score (mode), but does not reflect full consensus among participants.<br>👉 We invite you to consider this information in your new response.<br>📝 In the first round, you rated this indicator with a score of: X | 🔍 No clear consensus. 30% of experts rated this indicator with a 10, which represents the most frequent score (mode), but does not reflect full consensus among participants.<br>👉 We invite you to consider this information in your new response.<br>📝 In the first round, you rated this indicator with a score of: X | Comments were received from 3 experts. One expert considered this an ideal indicator, while others noted its low specificity for palliative care and its presence in official sources.<br><br>They mentioned potential overlap with more precise indicators related to pediatric palliative needs.<br><br>The experts suggest considering this indicator only as a complement, prioritizing those with greater thematic alignment. |
| In-Hospital Mortality Rate | Number of deaths occurring within hospitals compared with total hospitalizations | 🔍 No clear consensus. 44% of experts rated this indicator with a 10, which represents the most frequent score (mode), but does not reflect full consensus among participants.<br>👉 We invite you to consider this information in your new response.<br>📝 In the first round, you rated this indicator with a score of: X | 🔍 No clear consensus. 48% of experts rated this indicator with a 10, which represents the most frequent score (mode), but does not reflect full consensus among participants.<br>👉 We invite you to consider this information in your new response.<br>📝 In the first round, you rated this indicator with a score of: X | 🔍 No clear consensus. 33% of experts rated this indicator with an 8, which represents the most frequent score (mode), but does not reflect full consensus among participants.<br>👉 We invite you to consider this information in your new response.<br>📝 In the first round, you rated this indicator with a score of: X | Comments were received from 4 experts. The experts noted that this indicator may have potential if adjusted to identify palliative care–related cases.<br><br>They indicated that, as currently formulated, it does not reflect PHC integration nor distinguish between causes of death or palliative interventions. One expert considered it ideal.<br><br>The experts suggest reformulating the indicator to identify deaths plausible for PC and registered palliative interventions. |
| Index of Serious Health-Related Suffering in Children Aged 0–14 | Index of suffering from conditions amenable to palliative care in children aged 0–14 / Total deaths in 2019 | ✅ Strong consensus. 70% of experts rated this indicator with a 10.<br>👉 We invite you to consider this information in your new response.<br>📝 In the first round, you rated this indicator with a score of: X | 🔍 No clear consensus. 41% of experts rated this indicator with a 10, which represents the most frequent score (mode), but does not reflect full consensus among participants.<br>👉 We invite you to consider this information in your new response.<br>📝 In the first round, you rated this indicator with a score of: X | 🔍 No clear consensus. 56% of experts rated this indicator with a 10, which represents the most frequent score (mode), but does not reflect full consensus among participants.<br>👉 We invite you to consider this information in your new response.<br>📝 In the first round, you rated this indicator with a score of: X | Comments were received from 2 experts. The experts valued the indicator, but one expressed doubts about the measurement model used.<br><br>It was noted that the model may significantly influence need estimation and that reviewing its suitability for the pediatric PC context is important.<br><br>The experts suggest maintaining the indicator, reviewing the measurement model, and ensuring coherence with other approaches to pediatric palliative needs. |

✅ ¡Ready to submit!
