# Supplementary material for: Indicators for palliative care integration in primary health care: how expert profile shapes consensus in a modified Delphi study
Source: BMC Health Serv Res. 2026 Jun 8;26:1119. doi: 10.1186/s12913-026-14781-y (PMC13474756; doi:10.1186/s12913-026-14781-y)
Supplement: Supplementary file 2 [file 12913_2026_14781_MOESM9_ESM.pdf]

INFORMED CONSENT FORM

Design of a Synthetic Indicator for the Evaluation of Palliative Care Integration in the Colombian Health System

[Bogotá], [], [], []

Name of the test or procedure:

This project aims to design a synthetic indicator to evaluate the integration of palliative care in the Colombian health system. Your participation consists of being part of an expert panel that will carry out a Delphi method to select the partial indicators that will compose the synthetic indicator.

For data collection, you will be asked to record your opinions in an electronic form designed for this purpose. Completion of the Google Form will not be anonymous; however, your identity will not be disclosed under any circumstances. The processing of personal data will be conducted in accordance with Law 1581 of 2012 on the protection of personal data in Colombia.

I, [Name of the person authorizing participation in the test or procedure],

Identification document CC (   ) CE (   ) No. _________

This document is the property of the Faculty of Medicine, Universidad Antonio Nariño.
Reproduction is prohibited without authorization from the approving authority

Acting as (please select one of the following options):

( ) Autonomous participant, freely and voluntarily, in full exercise of my faculties.

I hereby declare that:

After being informed about the purposes, objectives, and procedures to be carried out during the research titled "Design of a Synthetic Indicator for the Evaluation of Palliative Care Integration in the Colombian Health System," and the possible risks that may arise from the procedure, I authorize my participation in the study, as well as the use of the data obtained for strictly academic and research purposes.

I also declare that I have been informed that:

My participation in this research is completely free and voluntary, and I may withdraw from it at any time.

I will not receive any personal benefit or financial compensation for participating in this project. However, the results obtained are expected to contribute positively to improving processes that may benefit people with conditions similar to mine, or those under my responsibility.

All information obtained and the results of this research will be treated confidentially; therefore, this information will be stored in paper and/or electronic format. The study files will be kept at Universidad Antonio Nariño, Circunvalar Campus, under the custody of the GRINCIBIO Research Group, affiliated with the Faculty of Medicine, and under the responsibility of the researchers participating in the project.

Since all information in this research project will be managed under anonymity, personal results will not be available to third parties such as employers, government organizations, insurance companies, the media, or other educational institutions. This also applies to the spouse, family members, and treating physicians (or other health professionals) of the participants.

This document is the property of the Faculty of Medicine, Universidad Antonio Nariño.
Reproduction is prohibited without authorization from the approving authority

In the event that my personal data, photographs, videos, or other information resulting from the implementation of the procedure or study are required for presentation for strictly academic or scientific purposes in events such as seminars, congresses, courses, symposia, clinical case reviews, or publications, among other types of scientific dissemination spaces, I authorize their use, if I so agree, by signing this document.

I declare that this document has been read and fully understood by me. Therefore, I acknowledge that I have been satisfactorily informed about the processes, procedures, or tests to be carried out by the professionals participating in the project as researchers, and I hereby give my consent.

In case of any questions, please contact the principal investigator Carolina Muñoz Olivar or the co-investigator Carlos Javier Avendaño at the following phone number: +XXX or by email camunoz97@uan.edu.co

Participant’s signature:

____

Participant’s signature

This document is the property of the Faculty of Medicine, Universidad Antonio Nariño.
Reproduction is prohibited without authorization from the approving authority

Principal Investigator and/or Co-investigators directly involved in the procedure or study:

____

Principal Investigator

____

Co-Investigator 1

____

Co-Investigator 2

This document is the property of the Faculty of Medicine, Universidad Antonio Nariño.
Reproduction is prohibited without authorization from the approving authority
